# The E3 Ubiquitin Ligases SINA3 and SINA5 Control DEP2 Ubiquitination and Proteasomal Degradation to Regulate Grain Size and Weight in Rice

**DOI:** 10.1111/pbi.70723

**Published:** 2026-07-17

**Authors:** Hongming Wu, Xiejun Sun, Xin Wang, Chen Xie, Yingli Lv, Xiaoman You, Yu Zhang, Xiaohang Han, Yun Zhu, Yimin Lin, Yehui Xiong, Qibing Lin, Cailin Lei, Xiuping Guo, Shanshan Zhu, Zhijun Cheng, Yulong Ren, Ling Jiang, Jianmin Wan

**Affiliations:** ^1^ National Key Laboratory of Crop Genetics & Germplasm Enhancement and Utilization Nanjing Agricultural University Nanjing China; ^2^ State Key Laboratory of Crop Gene Resources and Breeding, Institute of Crop Sciences Chinese Academy of Agricultural Sciences Beijing China; ^3^ Zhongshan Biological Breeding Laboratory Nanjing China

**Keywords:** CRISPR‐Cas9, E3 ligase, grain size, rice ( *Oryza sativa* L.), SINA, ubiquitination degradation

## Abstract

Grain size is a critical agronomic trait, yet the molecular mechanisms governing its determination in crops remain incompletely understood. While recent studies revealed that OsRING80 facilitates DENSE AND ERECT PANICLE 2 (DEP2) degradation to mediate immunity without impacting growth and development, our work identifies a distinct regulatory pathway controlling grain development. We demonstrate that two RING finger E3 ubiquitin ligases, SEVEN IN ABSENTIA 3 (SINA3) and SINA5, post‐translationally regulate the stability of DEP2 (also known as SRS1/EP2/OsRELA/SUG1) to modulate grain size. SINA3 and SINA5 physically interact with DEP2, specifically mediated through the C1 structural region of DEP2 containing a coiled‐coil domain. These E3 ligases promote K48‐linked polyubiquitination of DEP2, targeting it for proteasomal degradation. Liquid chromatography‐mass spectrometry (LC–MS) analysis identified six critical lysine residues (K399, K722, K746, K958, K962 and K1344) within DEP2 that are essential for its ubiquitylation and subsequent destabilization. Our genetic evidence further supports this regulatory module: knockout of *SINA3* and/or *SINA5* leads to DEP2 accumulation, concomitantly increasing grain size and 1000‐grain weight significantly, without altering other agronomic traits; conversely, overexpression of *SINA3* or *SINA5* reduces DEP2 protein levels and diminishes grain size and weight. Therefore, our study uncovers a novel post‐translational regulatory module where SINA3 and SINA5 control DEP2 stability to fine‐tune grain development. These findings present a promising strategy for optimizing grain yield by manipulating this post‐translational regulatory node.

## 1 Introduction

Rice ( *Oryza sativa* L.), a cornerstone of global food security (Xie et al. 2024; Xu et al. 2020), exhibits yield as a complex quantitative trait governed by multiple genetic factors (Xing and Zhang 2010). Critical determinants of yield include grain dimensions—length, width and thickness—along with grain filling rate, as these agronomic traits directly influence grain weight (Gasparis and Miloszewski 2023; Ren et al. 2023; Zhang et al. 2018). Extensive genetic studies have identified numerous genes and quantitative trait loci (QTLs) modulating grain size and weight (Gasparis and Miloszewski 2023; Ren et al. 2023). The encoded proteins function within diverse signalling networks, encompassing the ubiquitin‐proteasome pathway, G‐protein signalling, MAPK cascades, phytohormone pathways, transcriptional regulation and other independent or less‐defined mechanisms (Gasparis and Miloszewski 2023; Li and Li 2016). While many of these genes have been cloned and functionally characterized, the molecular mechanisms underpinning their regulation of grain size and weight, as well as their integration into comprehensive genetic networks, remain largely elusive.

Ubiquitin‐mediated processes are critically involved in regulating grain development (Huang et al. 2021; Shi et al. 2019; Song et al. 2007; Xia et al. 2013; Xie et al. 2024; Yuan et al. 2022). A key example is *GRAIN WIDTH 2 (GW2)*, the first cloned grain size QTL in rice, which encodes a cytoplasmic and nuclear RING‐type E3 ubiquitin ligase (Hao et al. 2021; Song et al. 2007). GW2 acts as a negative regulator of grain width by promoting the ubiquitination and degradation of its substrates, including expansin‐like 1 (EXPLA1) and the CC‐type glutaredoxin protein OsGRX8 encoded by *WIDE GRAIN 1* (*WG1*) (Choi et al. 2018; Garg et al. 2010; Hao et al. 2021). Conversely, *LARGE GRAIN 1* (*LG1*) encodes the deubiquitinating enzyme OsUBP15, positively influencing grain width by enhancing cell proliferation (Shi et al. 2019). The SEVEN IN ABSENTIA (SINA) family represents another class of highly conserved RING–type E3 ubiquitin ligase in both animals and plants (Den Herder et al. 2008; Sunnerhagen et al. 2003). Arabidopsis SINAT proteins are implicated in diverse processes, including hormone signalling, inflorescence development, abiotic stress responses and autophagy (Xia et al. 2020; Zhang et al. 2019). SINA proteins typically feature an N‐terminal RING domain responsible for ubiquitin ligase activity and a C‐terminal SINA‐type/TRAF domain facilitating protein–protein interactions and oligomerization (Polekhina et al. 2002; Xia et al. 2020; Xie et al. 2002). The rice genome encodes six SINA homologues (OsSINA1‐5 and OsDIS1) (Chang et al. 2022; Den Herder et al. 2008). Known rice SINA functions include OsRING80/OsDIS1, which regulates immune resistance by targeting DEP2 for degradation and modulates drought stress responses by controlling the stability of OsNek6 (Hou et al. 2025; Ning et al. 2011). Additionally, RMD interacting proteins 1–6 (RIP1‐6, SIAH‐type E3 ligases in rice) interact with RMD/OsFH5, influencing its subcellular localization; the *rip1‐6* sextuple mutant exhibits dwarfism, wrinkled seeds and wider leaves, phenocopying the *rmd* mutant (Chang et al. 2022; Yang et al. 2011). Most recently, rice SINA E3 ligases (SINARs) were shown to dichotomously regulate panicle morphogenesis and grain yield by mediating K63‐linked ubiquitination of rice ERECTA1 (OsER1), targeting it for degradation via the endosome‐to‐vacuole pathway (Lu et al. 2025). Despite these advances, the specific roles of individual SINA homologues and their cognate substrates in regulating plant growth and grain development remain largely unexplored.

The plant‐specific protein DEP2 (also known as SRS1, EP2, OsRELA, or SUB1) plays a critical role in regulating plant growth, panicle architecture and grain size (Abe et al. 2010; Li et al. 2010, 2025; Zhu et al. 2021, 2010). Loss‐of‐function mutants such as *dep2‐1*, *srs1* and *ep2* consistently exhibit characteristic phenotypes including short, erect panicles and small, round grains (Abe et al. 2010; Li et al. 2010; Zhu et al. 2010). Proposed molecular functions of DEP2 include acting as a transcriptional regulator. Evidence suggests OsRELA (Regulator of Leaf Angle 1) modulates leaf inclination by regulating the transcriptional activity of OsLIC (LEAF and TILLER ANGLE INCREASED CONTROLLER) (Zhu et al. 2021). Furthermore, SUG1 (Suppressor of the gain‐of‐function allele *GS2* ^*AA*^) functions downstream of GS2, potentially as a transcriptional regulator or cofactor, integrating signals from multiple transcription factors to control grain size through GA and BR signalling pathway, as well as broader growth pathways (Li et al. 2025). The stability of DEP2 is subject to post‐translational regulation. OsRING80 targets DEP2 for degradation, playing a crucial role in balancing plant immunity and growth (Hou et al. 2025). However, while OsRING80‐mediated degradation regulates DEP2 stability in the context of immunity, the specific mechanisms governing DEP2 protein stabilization during grain development remain to be elucidated.

Here, we obtained a novel allelic mutant of the *DEP2/SRS1* gene, designated *dep2‐3*, which exhibits distinct grain morphological characteristics compared to the previously reported *dep2‐1* and *dep2‐2* mutants (Li et al. 2010). Utilizing yeast two‐hybrid screening, we discovered that two RING finger E3 ubiquitin ligases, SINA3 and SINA5, interact with DEP2 and facilitate its degradation via the 26S proteasome‐dependent pathway. Notably, while OsRING80 is specifically involved in DEP2‐mediated immunity, *SINA3* and *SINA5* function as negative regulators of grain size and weight by mediating DEP2 degradation. Consistent with this mechanism, knockout of *SINA3* and/or *SINA5* (*sina3‐cr, sina5‐cr* single mutants and *sina3 sina5* double mutants) increased the stability of DEP2 protein in vivo, leading to significantly increased grain size and elevated 1000‐grain weight. Collectively, our findings reveal a critical post‐translational pathway that modulates DEP2 stability to regulate grain development, offering novel targets for yield improvement in rice.

## 2 Results

### 2.1 SINA3 and SINA5 Physically Interact With DEP2

Previous studies established that *DEP2* regulates plant growth, panicle architecture and grain size (Abe et al. 2010; Li et al. 2010). In this study, we characterized a novel allelic mutant of *DEP2/SRS1*, designated *dep2‐3* (Hou et al. 2025). To elucidate the mechanisms regulating DEP2 protein stability during grain development, we conducted a yeast two‐hybrid (Y2H) screen for DEP2‐interacting partners. This screen identified the RING finger E3 ubiquitin ligase SINA5 as a putative DEP2 interactor (Figure S1a,b). The rice genome encodes six SINA homologues (Figures S1c and S2a,b), and subsequent pairwise Y2H assays confirmed specific interactions between DEP2 and both SINA3 and SINA5 (Figure S1c). Given prior evidence from *Arabidopsis*, human and *M. truncatula* studies indicating that E3 ligases can form dimers (Den Herder et al. 2008; Xia et al. 2020; Xie et al. 2002). We further investigated SINA oligomerization in rice. Y2H assays revealed that rice SINA proteins are capable of forming both homomeric and heteromeric complex (Figure S2c).

To elucidate the cellular functions of SINA3/5 proteins in rice, we first determined their subcellular localization. Therefore, we generated constructs encoding SINA3 and SINA5 fused to the C‐terminal of GFP (SINA3‐GFP and SINA5‐GFP) for transient expression in wild‐type rice protoplasts. Both SINA3‐GFP and SINA5‐GFP fusion proteins exhibited a punctate localization pattern within the cytoplasm and nucleus (Figure S3). As a control, fluorescence from the empty GFP vector was distributed throughout the cytoplasm and nucleus (Figure S3a). DEP2, a plant‐specific protein, localizes to multiple organelles, including the cytoplasm, plasma membrane and nucleus (Li et al. 2010, 2025; Zhu et al. 2021). Notably, upon co‐expression of SINA3‐GFP or SINA5‐GFP with DEP2‐mCherry in *N. benthamiana* leaf epidermal cells, both fusion proteins displayed a high degree of colocalization with DEP2‐mCherry in the nucleus and cytoplasm (Figure S4).

**FIGURE 1:**
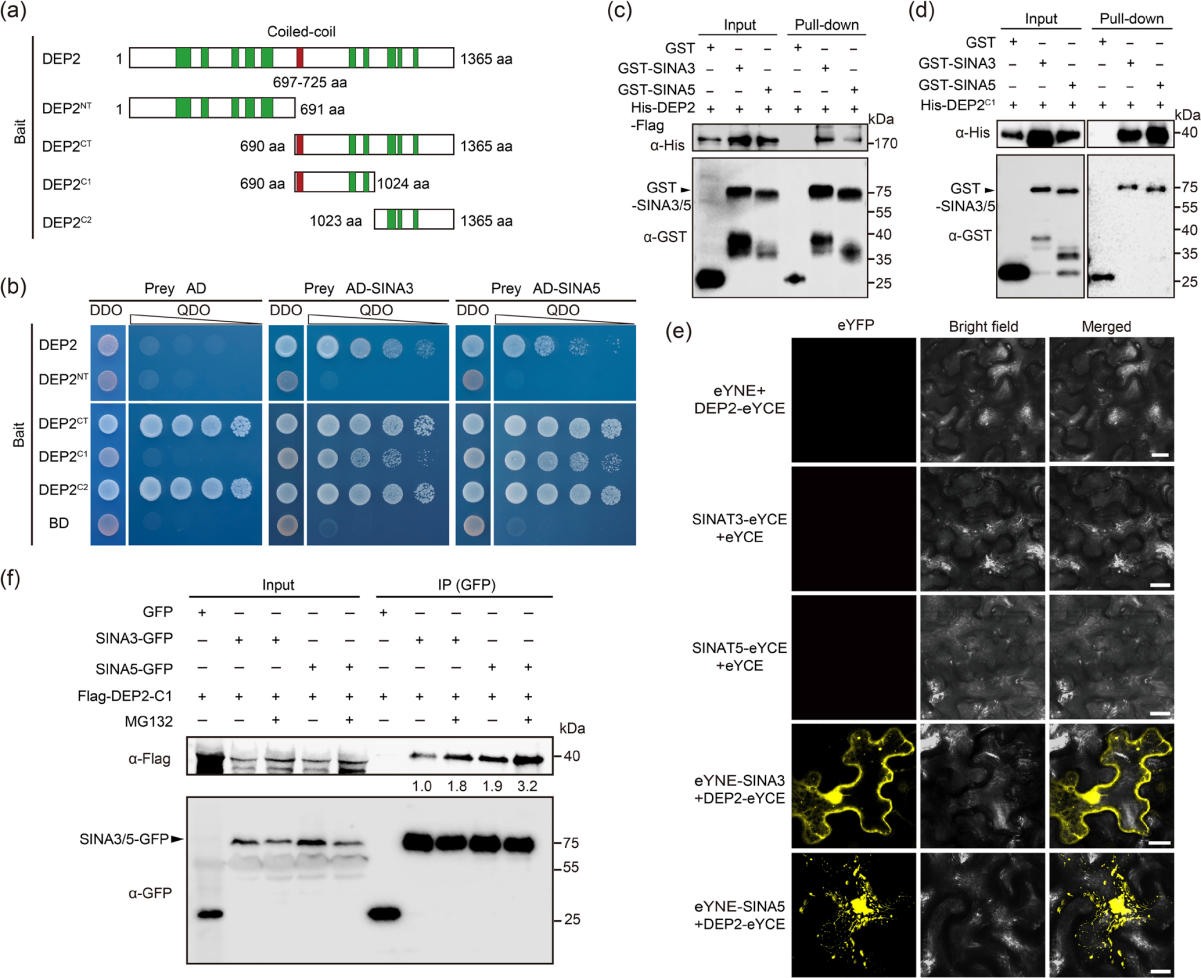
DEP2 interacts with the E3 ubiquitin ligases SINA3 and SINA5. (a) Schematic diagrams of constructs for Y2H assay. DEP2, DEP2^NT^, DEP2^CT^, DEP2^C1^ and DEP2^C2^ represent the full‐length DEP2 protein and different truncated fragments. aa, amino acids. Domain structure of DEP2 protein: Coiled‐coil (697–725 aa, red). Disordered region (enriched in basic/acidic/polar residues, green). (b) Y2H assay reveals that full‐length DEP2 and truncated protein DEP2^C1^ interact with SINA3/5. Transformed yeast cells were spotted on the control medium DDO (SD/−Trp/−Leu) and selective medium QDO (SD/−Trp/−Leu/‐His/−Ade). (c, d) In vitro pull‐down assay validates the interaction between SINA3/5 and DEP2. Induced His‐DEP2 and His‐DEP2^C1^ were incubated with the glutathione S‐transferase (GST)‐SINAs or GST protein. (e) BiFC assay confirms the interactions between DEP2 and SINA3/5. Full‐length *SINA3* and *SINA5* were fused with nYFP and coexpressed with cYFP‐fused DEP2 in the leaf epidermal cells of *N. benthamiana* treated with 50 μM MG132. Bars = 10 μm. (f) Co‐immunoprecipitation assay showing that DEP2^C1^ interacts with SINA3/5 in *N. benthamiana* leaves. “+” and “–” indicate the presence and absence of the components in each reaction mixture, respectively.

*DEP2* encodes a large protein of 1365 amino acid residues, containing only a predicted coiled‐coil domain within its central region. To identify the specific DEP2 domain mediating interaction with SINA3/5, we generated two truncation constructs: an N‐terminal region DEP2^NT^ and a C‐terminal fragment DEP2^CT^ (Figure 1a). The DEP2^CT^ fragment encompasses the coiled‐coil domain, a structural motif known to facilitate protein–protein interactions (Burkhard et al. 2001). Y2H assays revealed that DEP2^NT^ failed to interact with SINA3/5, while DEP2^CT^ exhibited self‐activation (Figure 1b). To circumvent this, we subdivided DEP2^CT^ into two smaller fragments, DEP2^C1^ and DEP2^C2^. Subsequent Y2H analysis demonstrated that DEP2^C1^ strongly interacted with SINA3/5, whereas DEP2^C2^ still displayed self‐activation (Figure 1a,b). Furthermore, in vitro GST pull‐down assays confirmed these interactions: both GST‐SINA3 and GST‐SINA5, but not GST alone, efficiently pulled down full‐length DEP2 and the DEP^C1^ truncation (Figure 1c,d). We also verified the SINA3/5‐DEP2 interaction in vivo using BiFC and Co‐IP assays in *N. benthamiana* leaves (Figure 1e,f). Notably, treatment with the proteasome inhibitor MG132 enhanced the SINA3/5‐DEP2^C1^ interaction, as evidenced by increased enrichment of Flag‐DEP2^C1^ by GFP beads in Co‐IP experiments (Figure 1f). Collectively, these results demonstrate that SINA3/5 physically interact with DEP2, primarily through the DEP2 C1 domain.

### 2.2 SINA3 and SINA5 Ubiquitinate and Stabilize DEP2 In Vitro

**FIGURE 2:**
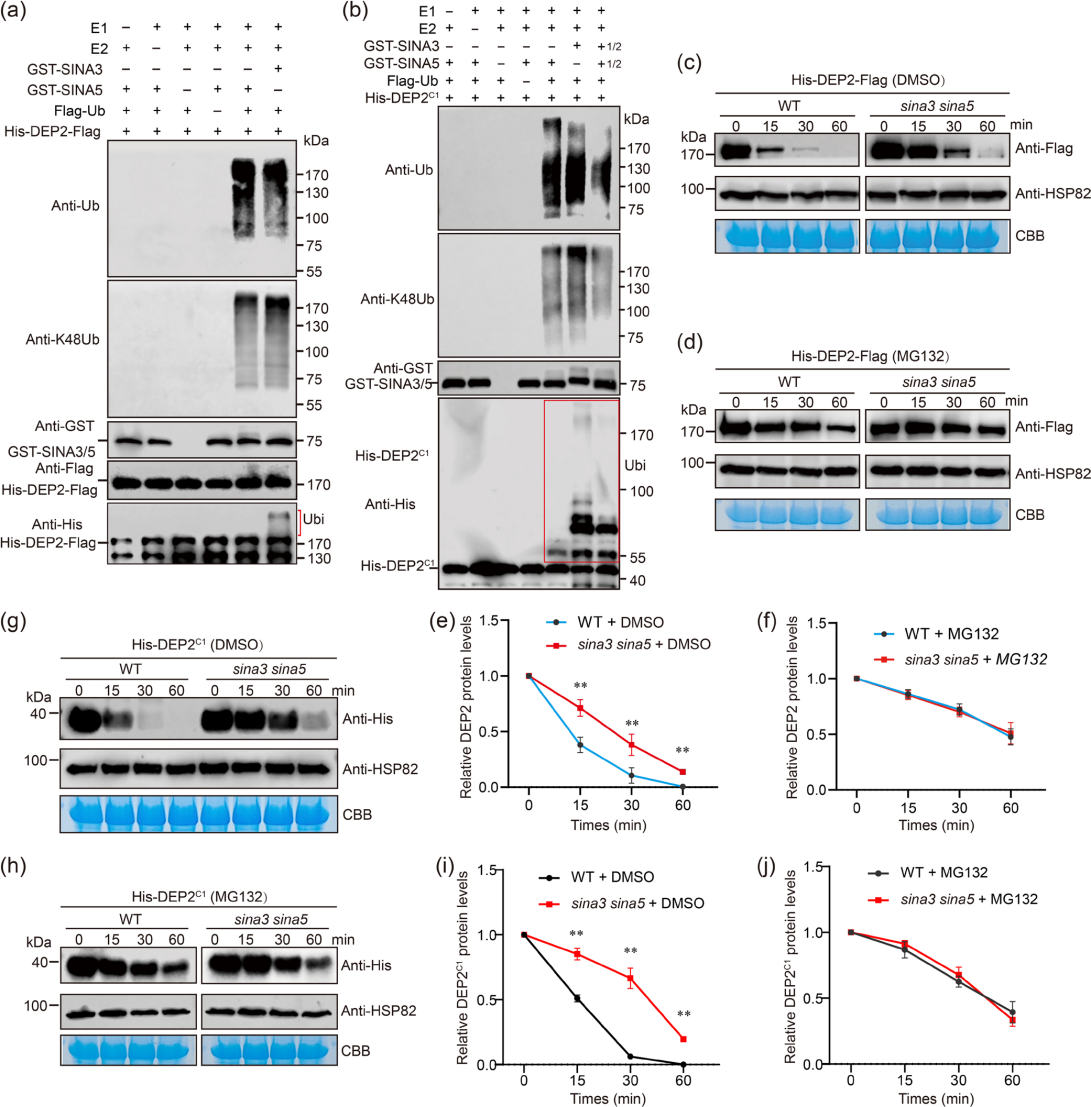
SINA3 and SINA5 ubiquitinate DEP2 and modulate its abundance. (a, b) In vitro ubiquitination of DEP2 (a) and DEP2^C1^ (b) by GST‐SINA3 and GST‐SINA5. Flag‐tagged Ubiquitin (Ub) proteins (Flag‐Ub), GST‐SINA3 or GST‐SINA5 and His‐DEP2‐Flag or His‐DEP2^C1^ were added to reactions in the presence of ATP. DEP2 polyubiquitination was detected by immunoblotting with an anti‐His, anti‐Ub and anti‐K48Ub antibodies. Red box indicates polyubiquitinated protein. “+” and “–” indicate the presence and absence of the components in each reaction mixture, respectively. “+ 1/2” indicates adding half the amount of GST‐SINA3 or GST‐SINA5. (c, d) Cell‐free protein degradation assay in vitro. Total proteins extracted from transgenic lines of the *sina3 sina5* double mutants and WT were treated with or without 100 μM MG132, and then incubated with the purified His‐DEP2‐Flag fusion protein. The samples were collected at different time points. HSP82 was used as an internal reference. CBB, Coomassie Brilliant Blue, represents the Rubisco protein. (e) Quantification of His‐DEP2‐Flag protein level in (c). Values are means ± SD from three independent experiments (Student's *t*‐test, **p < 0.01**). (f) Quantification of His‐DEP2‐Flag protein level in (d). (g, h) Cell‐free protein degradation assay in vitro. Total proteins extracted from transgenic lines of the *sina3 sina5* double mutant and WT were treated with or without 100 μM MG132, and then incubated with the purified His‐DEP2^C1^ fusion protein. (i) Quantification of His‐DEP2^C1^ protein level in (g). (j) Quantification of His‐DEP2^C1^ protein level in (h). Values are means ± SD from three independent experiments (Student's *t*‐test, **p < 0.01**).

To investigate the ubiquitination activity of SINA3/5 on DEP2 protein, we performed in vitro ubiquitination assays. Consistent with their predicted E3 ligase function and the presence of a RING zinc finger domain (Chang et al. 2022; Ning et al. 2011), GST‐SINA3 and GST‐SINA5 exhibited autoubiquitination activity in reactions containing an E1 ubiquitin‐activating enzyme, an E2 ubiquitin‐conjugating enzyme, and His‐tagged ubiquitin (His‐Ub) (Figures S5 and S6). Using the same ubiquitination system, we found that both GST‐SINA3 and GST‐SINA5 efficiently ubiquitinated full‐length DEP2 and the DEP2^C1^ fragment (Figure 2a,b; Figure S5). Since K48**–**linked polyubiquitin chains typically target substrates for 26S proteasomal degradation (Nathan et al. 2013; Yau and Rape 2016), we performed immunoblot analysis with an anti‐K48‐linked ubiquitin antibody. Distinct high molecular‐weight laddering patterns confirmed that His‐DEP2‐Flag and His‐DEP2^C1^ undergo SINA3/5‐mediated K48‐linked polyubiquitination (Figure 2a,b). In contrast, no K63‐linked ubiquitination of these substrates was detected (Figure S7), demonstrating that SINA3/5 specifically mediate K48‐linked ubiquitination of DEP2 in vitro. Comparative analysis revealed that GST‐SINA3 exhibited stronger ubiquitination activity toward both His‐DEP2‐Flag and His‐DEP2^C1^ than GST‐SINA5, as indicated by more intense ubiquitin laddering detected with both anti‐K48Ub and anti‐His antibodies (Figure 2a,b). Intriguingly, when GST‐SINA3 and GST‐SINA5 were added simultaneously to mediate the ubiquitination of His‐DEP2^C1^, SINA3 and SINA5 formed a heterodimer. This heterodimer exhibited moderate ubiquitination activity toward DEP2, with an activity level intermediate between those of SINA3 and SINA5 alone (Figure 2b). This attenuation may indicate that heterodimer formation between SINA3 and SINA5 may modulate their E3 ligase activity.

To determine whether the E3 ubiquitin ligase SINA3/5 regulate DEP2 stability, we conducted cell‐free protein degradation assays. Recombinant His‐DEP2‐Flag protein was incubated with equal amounts of total protein extracts derived from either wild‐type rice seedlings or the *sina3 sina5* double mutant. Immunoblot analysis using an anti‐Flag antibody revealed that the degradation of His‐DEP2‐Flag was significantly slower in extracts lacking functional SINA3/5 (*sina3 sina5*) compared to WT extracts (Figure 2c,e). Furthermore, addition of the 26S proteasome inhibitor MG132 to WT extracts reduced the degradation rate of His‐DEP2‐Flag to a level comparable to that observed in the *sina3 sina5* extracts (Figure 2d,f). Consistent with these findings, depletion of *SINA3/5* similarly stabilized the truncated DEP2^C1^ fragment (Figure 2g–j). Collectively, these results demonstrate that SINA3/5 function as E3 ubiquitin ligases targeting DEP2 for ubiquitination and subsequent degradation via the 26S proteasome pathway.

### 2.3 SINA3 Ubiquitinates DEP2 on the C1 Domain and Six Lys Sites Contribute to DEP2 Ubiquitylation

**FIGURE 3:**
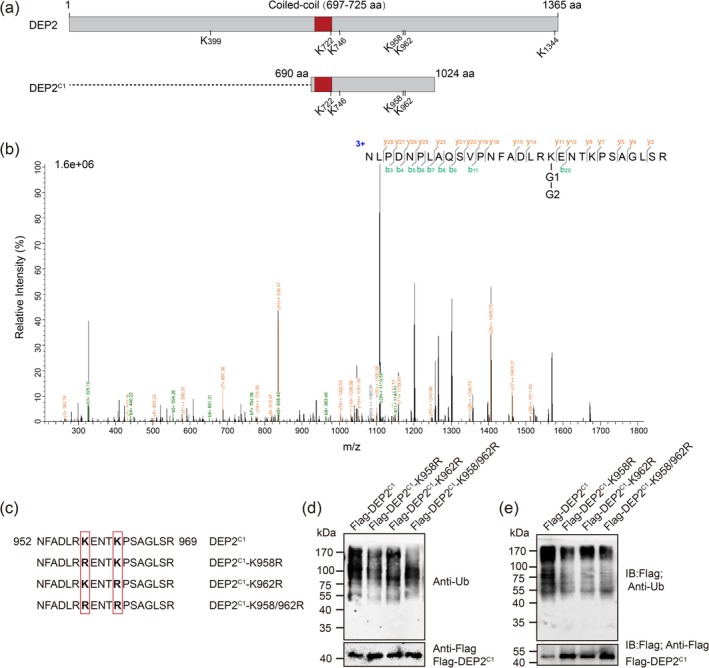
Identification of the ubiquitination sites on DEP2. (a) Identification of ubiquitination sites in the full‐length DEP2 protein and truncated fragments DEP2^C1^ using liquid chromatography‐mass spectrometry (LC–MS). Red box represents coiled‐coil domain (697–725). The Lys (K) residues marked with numbers, which were identified by LC–MS, could be ubiquitinated by SINA3 in vitro. The K399, K722, K746, K958, K962 and K1344 Lys sites contribute to DEP2 ubiquitylation. (b) K958 in the DEP2^C1^ peptide NLPDNPLAQSVPNFADLRKENTKPSAGLSR was ubiquitinated. The theoretical MW is 3362.7365, *z* is 3, and *m*/*z* = 1121.9194. Two glycines from ubiquitin were found on lysine in this peptide. (c) Alignment of DEP2^C1^ and the DEP2^C1^ ubiquitylation site mutant. DEP2^C1^‐K958R indicates the K → R substitutions of K958, DEP2^C1^‐K962R indicates the K → R substitutions of K962, and DEP2^C1^‐K958/962R indicates both the K958 and K962 point substitutions to Arg (R, the abbreviation of arginine acid). (d, e) DEP2^C1^ levels in the ubiquitylation site mutants. Flag‐tagged DEP2^C1^ (Flag‐DEP2^C1^) and DEP2^C1^ ubiquitylation site mutants (Flag‐DEP2^C1^‐K958R, Flag‐DEP2^C1^‐K962R and Flag‐DEP2^C1^‐K958/962R) were expressed in wild‐type protoplast. Total protein was extracted after incubation for 14 h under constant 25°C conditions and immunoprecipitated by anti‐Flag beads. The samples were collected before (d) and after (e) immunoprecipitation. The blots were probed with anti‐Flag and anti‐Ub antibodies. Numbers on the left indicate molecular mass (kDa) of each band.

Our biochemical data demonstrate that SINA3/5 interact with DEP2 specifically via its C1 domain (Figure 1). Furthermore, in vitro ubiquitination assays revealed that SINA3/5 targets the DEP2^C1^ fragment for K48‐linked polyubiquitination and proteasomal degradation, similar to full‐length DEP2 (Figure 2). Based on these findings, we hypothesized that the DEP2 C1 domain serves as both the interaction interface for SINA3/5 and the primary site for polyubiquitination. To identify ubiquitination sites, we performed liquid chromatography‐mass spectrometry (LC–MS) on in vitro ubiquitination reactions catalysed by GST‐SINA3. This analysis identified six ubiquitinated lysine residues (K399, K722, K746, K958, K962 and K1344) in full‐length DEP2 (Figure 3a; Figure S8). Notably, four of these sites (K722, K746, K958 and K962) were also detected within the DEP2^C1^ fragment (Figure 3a,b). These results confirm that SINA3 directly ubiquitinates DEP2 within the C1 domain, consistent with its role as the primary interaction and regulatory module for these E3 ligases.

To evaluate the functional significance of the identified ubiquitination sites, we generated site‐directed mutants by substituting lysine residues K958 and K962 with arginine (R) in DEP2^C1^. This yielded three constructs: Flag‐DEP2^C1^‐K958R, Flag‐DEP2^C1^‐K962R and Flag‐DEP2^C1^‐K958/962R (Figure 3c). We then transiently expressed these mutants alongside wild‐type Flag‐DEP2^C1^ in rice protoplasts to assess their impact on ubiquitination and protein stability. Immunoblot analysis revealed significantly reduced ubiquitylation of Flag‐DEP2^C1^ in both K958R and K962R single mutants compared to wild‐type controls. Ubiquitination was further diminished in the K958/962R double mutant (Figure 3d,e), indicating that K958 and K962 are critical ubiquitylation sites. Consistent with these ubiquitylation defects, Flag‐DEP2^C1^ protein accumulation increased progressively in single and double mutants (Figure 3d). These results demonstrate that K958 and K962 within the DEP2 C1 domain are essential for efficient polyubiquitination. Combined with our earlier findings, this confirms that the C1 domain serves as both the primary interaction interface for SINA3/5 and the major target for their E3 ligase activity.

### 2.4 Function Loss of *SINA3/5* Promotes Cell Expansion and Thus Increases Grain Size

**FIGURE 4:**
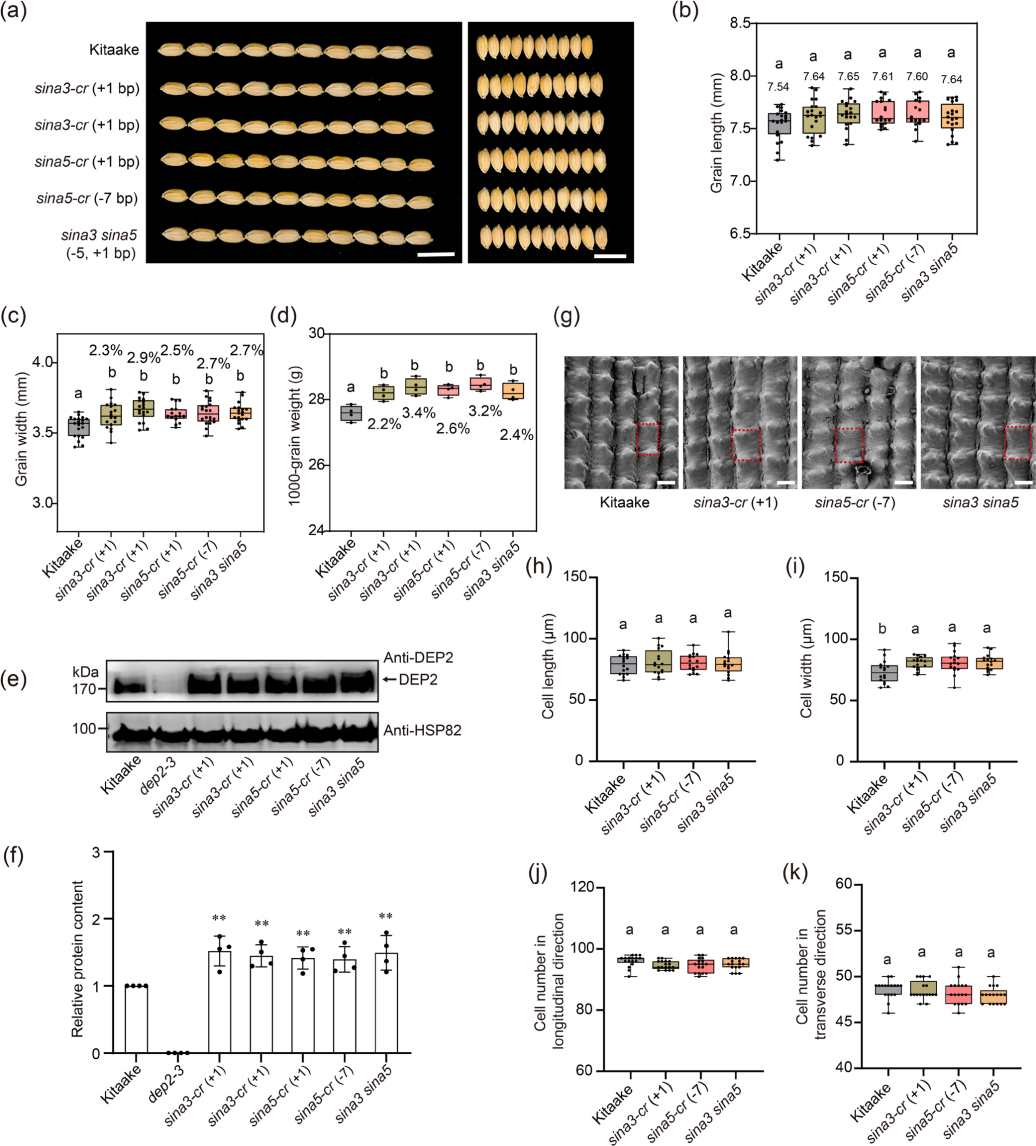
Knockout of *SINA3* and *SINA5* in the Kitaake background. (a) Grain morphologies of Kitaake, *sina3‐cr*, *sina5‐cr* and *sina3 sina5* transgenic lines. Bars = 1 cm. (b–d) Comparisons of the grain length (b), grain width (c) and 1000‐grain weight (d) of Kitaake, *sina3‐cr*, *sina5‐cr* and *sina3 sina5*. Values are means ± SD (*n* ≥ 18 in b, c and *n* ≥ 3 in d). Different letters indicate significant differences ranked by pairwise multiple comparison followed by Tukey's test (*p* < 0.05). (e) Protein levels of DEP2 in the overexpression plants of Kitaake, *dep2‐3*, *sina3‐cr*, *sina5‐cr* and *sina3 sina5* mutants. HSP82 was used as a loading control. (f) The grayscale values of protein bands for DEP2 (e) were measured. Values are means ± SD (Four biological replicates). Asterisks indicate significant differences compared with the wild type, according to Student's *t*‐test (**p < 0.01). (g) SEM analysis of the lemmas of Kitaake, *dep2‐3*, *sina3‐cr* (+1), *sina5‐cr* (−7) and *sina3 sina5*. The red dashed box represents a cell of the lemmas. Bar = 50 μm. (h–k) Average cell length (h), cell width (i), cell number in the longitudinal direction (j) and in the transverse direction (k) of lemmas cells in Kitaake, *dep2‐3*, *sina3‐cr* (+1), *sina5‐cr* (−7) and *sina3 sina5*. Values are means ± SD (*n* ≥ 12). Different letters indicate significant differences ranked by pairwise multiple comparison followed by Tukey's test (*p* < 0.05).

Given the established SINA3/5‐DEP2 interaction and SINA3‐mediated ubiquitination of DEP2, we hypothesized that SINAs regulate grain size by controlling DEP2 stability. To test this, we generated *SINA3* and *SINA5* single mutants (*sina3‐cr*, *sina5‐cr*) and a *sina3 sina5* double mutant using CRISPR‐Cas9 (Figure S9). Phenotypic analysis revealed that all mutants exhibited statistically significant increases in grain width and 1000‐grain weight relative to wild‐type controls (Figure 4a–d). In these mutants, grain length exhibited a slight but non‐significant increase (Figure 4b), whereas grain thickness, plant height and other agronomic traits showed no significant changes (Figure 4a–d; Figure S10; Table S1). Consistent with the phenotypic observations, immunoblot analysis demonstrated significantly elevated DEP2 protein accumulation in both single and double mutants compared to WT (Figure 4e,f; Figure S11). Furthermore, scanning electron microscopy (SEM) of mutant glumes revealed a pronounced increase in cell width–aligning with the enhanced grain width phenotype–while cell length and longitudinal/transverse cell numbers were comparable to WT (Figure 4g–k).

In addition, we performed quantitative reverse transcription PCR (RT‐qPCR) to investigate the expression levels of *SINA3* and *SINA5* in the respective genetic backgrounds, and found that the transcript level of *SINA3* was downregulated in the *sina3‐cr* single mutant, whereas the expression of *SINA5* was significantly upregulated (Figure S12a). Similarly, in the *sina5‐cr* single mutant, the expression level of *SINA5* was significantly reduced, while the expression level of *SINA3* showed a notable increase (Figure S12b). In the *sina3 sina5* double mutant, the expression levels of *SINA3* and *SINA5* both decreased (Figure S12). Both SINA3 and SINA5 physically interact with DEP2 and are involved in its degradation, suggesting that they perform functionally redundant roles, thereby conferring phenotypic compensation in the respective single mutants. Together, these findings indicate that SINA3 and SINA5 cooperatively regulate grain size by tuning DEP2 protein abundance to modulate cell expansion.

### 2.5 SINA3/5 Ubiquitinates DEP2 to Control Grain Size in Rice

DEP2 plays a critical role in regulating panicle architecture and grain size (Li et al. 2010; Zhu et al. 2010). The RT‐qPCR analysis revealed that *DEP2* was broadly expressed in all examined tissues, with its expression gradually increasing during spikelet development, peaking at the 6 cm spikelet stage, and then declining (Figure S13a). As E3 ubiquitin ligases that regulate the protein stability of DEP2, *SINA3* and *SINA5* exhibit distinct expression patterns compared with *DEP2*. RT‐qPCR analysis showed that *SINA3* expression was relatively low during early spikelet development but continued to increase in subsequent developmental stages (Figure S13b). In addition, *SINA5* expression level reaches its peak at the 8 cm spikelet stage, later than that of DEP2 (Figure S13b). This indicates that *SINA3/5* are expressed later than *DEP2*, and when spikelet development reaches a specific stage or nears completion, SINA3/5 exerts a braking effect on DEP2 to negatively regulate its function.

**FIGURE 5:**
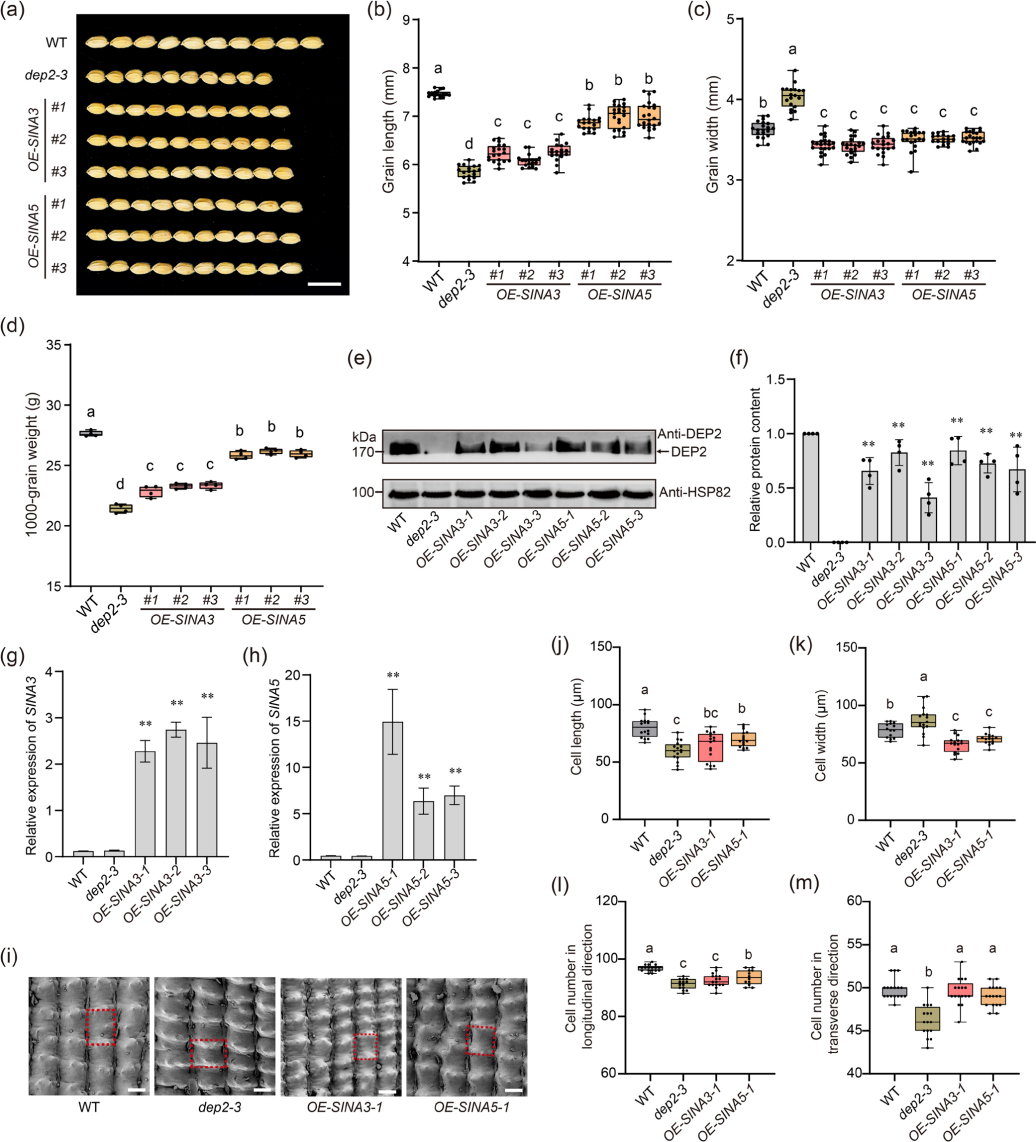
SINA3 and SINA5 function by ubiquitinating DEP2 to control grain size. (a) Grain morphologies of wild type (WT), *dep2‐3*, *OE‐SINA3* (−1 and −2) and *OE‐SINA5* (−1 and −2) transgenic lines. Bars = 1 cm. (b–d) Comparisons of the grain length (b), grain width (c) and 1000‐grain weight (d) of WT, *dep2‐3, OE‐SINA3* and *OE‐SINA5*. Values are means ± SD (*n* ≥ 20 in b, c and *n* = 3 in d). Different letters indicate significant differences ranked by pairwise multiple comparison followed by Tukey's test (*p* < 0.05). (e) Protein levels of DEP2 in the overexpression plants of *OE‐SINA3* and *OE‐SINA5*. HSP82 was used as a loading control. (f) The grayscale values of protein bands for DEP2 (e) were measured. Values are means ± SD (Four biological replicates). Asterisks indicate significant differences compared with the wild type, according to Student's *t*‐test (*p < 0.05, **p < 0.01). (g, h) Relative *SINA3* and *SINA5* transcript levels in spikelet hulls of wild type, *dep2‐3* and the overexpression plants of *OE‐SINA3* and *OE‐SINA5*. Rice *UBIQUITIN* gene was used as an internal control. Values are means ± SD (*n* = 3). Asterisks indicate significant differences compared with the wild type, according to Student's *t*‐test (**p < 0.01). (i) SEM analysis of the lemmas of WT, *dep2‐3*, *OE‐SINA3*‐1 and *OE‐SINA5*‐1. The red dashed box represents a cell of the lemmas. Bar = 50 μm. (j–m) Average cell length (j), cell width (k), cell number in the longitudinal direction (l) and in the transverse direction (m) of lemmas cells in WT, *dep2‐3*, *OE‐SINA3*‐1 and *OE‐SINA5*‐1. Values are means ± SD (*n* ≥ 12). Different letters indicate significant differences ranked by pairwise multiple comparison followed by Tukey's test (*p* < 0.05).

To further investigate the negative regulatory effect of SINA3/5 on DEP2, we overexpressed *SINA3* and *SINA5* in Kitaake and found that three positive transgenic lines produced smaller grains compared with the wild type (Figure 5a–h; Table S2). Phenotypic analysis revealed that the plant height, panicle length, grain length, grain width and 1000‐grain weight of *OE‐SINA3* and *OE‐SINA5* lines significantly reduced, yet remained higher than those of the DEP2 functional deletion mutant *dep2‐3* (Figure 5b,d; Table S2). The *OE‐SINA3* lines displayed a more substantial decrease in grain length and 1000‐grain weight compared with the *OE‐SINA5* lines; it seemed that *SINA3* exhibited a stronger detrimental effect on grain development than *SINA5* (Figure 5a–d). Supporting this notion, immunoblot analysis with the anti‐DEP2 antibodies showed that the *SINA3* overexpression more dramatically reduced the abundance of DEP2 protein than the *SINA5* overexpression (Figure 5e,f; Figure S11). Furthermore, we overexpressed *SINA5* in the *dep2‐3* mutant background and obtained three independent overexpression lines. The results showed that these lines exhibit the same grain phenotypes as the *dep2‐3* mutant (Figure S14), suggesting that *DEP2* functions downstream of *SINA5*.

We next analysed epidermal cells of mature grain lemmas in WT, *dep2‐3*, *OE‐SINA3* and *OE‐SINA5* lines (Figure 5i–m). Both *OE‐SINA3* and *OE‐SINA5* lines exhibited significantly reduced glume cell length compared to WT—a phenotype mirroring the *dep2‐3* mutant (Figure 5i,j). Similarly, glume cell width decreased in overexpression lines relative to WT (Figure 5k). Longitudinal cell number was significantly reduced in *SINA*‐overexpressing lemmas, while transverse cell number remained unchanged (Figure 5l,m). These cellular alterations demonstrate that SINA3/SINA5 restrict grain growth by coordinately suppressing cell expansion (length/width) and longitudinal cell proliferation within the spikelet hull. Collectively, our genetic and biochemical evidence establishes that SINA3/5 regulate grain size and weight through ubiquitin‐mediated degradation of DEP2 in rice.

## 3 Discussion

Grain size and weight are critical yield determinants in cereal crops (Bai et al. 2023; Lyu et al. 2020; Qiao et al. 2021; Xie et al. 2024). Understanding the molecular mechanisms governing grain size regulation is therefore essential for yield enhancement. Previous studies have established that DEP2/SRS1 modulates grain size and weight by regulating cell expansion and proliferation in the spikelet hull (Abe et al. 2010; Li et al. 2010). Furthermore, DEP2/SUG1 integrates with multiple transcription factors to coordinate grain development through GA and BR signalling pathways, as well as growth‐related cascades (Li et al. 2025). Notably, OsRING80 interacts with DEP2 and promotes its degradation via the 26S proteasome pathway, but functions specifically in immunity without affecting grain development (Hou et al. 2025). Consequently, the post‐translational regulatory mechanisms underlying DEP2's role in grain size and weight determination remain poorly characterized.

Our study elucidates that SINA3 and SINA5 function as key regulators of grain size by controlling DEP2 stability, thereby defining a pathway distinct from the immune‐related OsRING80‐DEP2 module. Overexpression of *SINA3* and *SINA5* markedly reduced DEP2 protein abundance and impaired key agronomic traits, including plant height, panicle length, grain size and 1000‐grain weight, compared to the wild type controls (Figure 4c; Table S2). Moreover, SEM analysis revealed that SINA3, SINA5 and DEP2 coordinately regulate grain size by modulating both cell proliferation and expansion. Lemmas of *dep2‐3*, *OE‐SINA3*‐1 and *OE‐SINA5‐1 lines* exhibited significantly fewer longitudinal cells, with cell lengths reduced by 25.36%, 20.04% and 12.12%, respectively, relative to WT (Figure 5i–m). These results indicate that restricted cell expansion is the primary factor underlying smaller grains in *OE‐SINA3‐1* and *OE‐SINA5‐1* lines, consistent with phenotypes observed in *dep2‐1*, *dep2‐2*, *dep2‐3* and *srs1* mutants (Abe et al. 2010; Li et al. 2010; Hou et al. 2025). Collectively, SINA3/5 act as negative regulators of grain development by coregulating cell expansion and proliferation in the spikelet hull. Notably, *OE‐SINA3* and *OE‐SINA5* lines also displayed significant reductions in plant height and panicle length (Table S2), indicating SINA3/5 function extends beyond grain development to broader plant growth processes. These findings further support that SINA3/5 and DEP2 operate within a common genetic pathway.

Interestingly, in contrast to the significant reduction in grain length in *OE‐SINA3* and *OE‐SINA5* lines, grain length displayed a slight but non‐significant increase in the *sina3‐cr*, *sina5‐cr* single mutants and *sina3 sina5* double mutant (Figure 4b). This phenotypic asymmetry suggests the existence of a compensatory mechanism between SINA3 and SINA5. Both SINA3 and SINA5 physically interact with DEP2 and mediate its degradation, and mutation of one gene leads to markedly increased expression of the other (Figure 2; Figure S12), indicating that these two genes exert a certain compensatory effect. Consistently, cell‐free protein degradation assays found that the *sina3 sina5* double mutant significantly reduced the degradation rate of DEP2 in vitro, but did not completely block its degradation (Figure 2c), implying that other E3 ubiquitin ligases are also involved in regulating DEP2 protein stability. Such compensatory effects reduced the impact of *SINA3* and *SINA5* mutations on the grain phenotype. In contrast, overexpression of *SINA3* or *SINA5* strongly promoted DEP2 degradation, reducing its protein abundance (Figure 5e,f). The content of the DEP2 protein may be below a functional threshold and thereby severely compromising DEP2 activity. This explains why overexpression of *SINA3* or *SINA5* led to a dramatic reduction in grain length, whereas mutation of SINA3/5 conferred only a limited increase.

Ubiquitin proteasome‐dependent protein degradation is a rapid, selective regulatory mechanism that plays critical roles in diverse cellular processes, including grain development (Hao et al. 2021; Xie et al. 2024; Xu and Xue 2019; Xu et al. 2024; Yang et al. 2021). OsOTUB1 interacts with OsSPL14 (SQUAMOSA PROMOTER BINDING PROTEIN‐LIKE14) to inhibit its K63‐ubiquitination, which in turn promotes K48Ub‐dependent proteasomal degradation of OsSPL14 (Wang et al. 2017). The RING E3 ligase CLG1 (Chang Li Geng 1) targets GS3 and mediates K63‐linked polyubiquitination, thereby promoting GS3 degradation through the endosomal pathway to regulate grain size in rice (Yang et al. 2021). Unlike SINARs, which mediate K63‐linked ubiquitination of OsER1 (Lu et al. 2025), our data suggested that SINA3/5 catalyse K48‐linked ubiquitination of DEP2. Furthermore, the truncated C1 domain is a specific structural domain for the interaction between DEP2 and SINA3/5. The LC–MS analysis further identified four ubiquitination sites (K722, K746, K958 and K962) within the C1 domain, which contains the majority of six ubiquitin sites of the full‐length DEP2 protein (Figure 3). These results suggested that the C1 domain of DEP2 was identified to mediate the interaction with SINA3/5, and this domain is mainly subjected to polyubiquitination. The K399 and K1344 of DEP2 in the non‐interacting binding region can also be ubiquitinated by SINA3. The presence of additional ubiquitination sites outside the interaction region suggests a potentially more complex regulatory pattern, possibly involving spatial conformation of the full‐length DEP2 protein. Future investigations into the structural characteristics of DEP2 may shed light on these unresolved issues, and whether DEP2 is subject to other stable regulatory mechanisms warrants additional research.

In plants, SINA homologues participate in diverse growth, stress response and immunity processes (Hou et al. 2025; Nolan et al. 2017; Qi et al. 2022; Xie et al. 2002; Zhang et al. 2019). In Arabidopsis, SINATs mediated the ubiquitination and degradation of FREE1 and VPS23A and contributed to the modulation of ABA signalling. Furthermore, the SINAT‐FREE1/VPS23A proteins were co‐degraded by the vacuolar pathway (Zhang et al. 2019). In *M. domestica*, MdRGL2a and MdCIPK20 are ubiquitinated and degraded by MdSINA1 and MdSINA2, respectively, via the proteasome pathway, which contributes to the inhibition of GA on anthocyanin accumulation (An et al. 2023). In rice, the SEVEN IN ABSENTIA of RICE (SINAR) family contributes either synergistically or antagonistically to rice spikelet number by targeting OsER1 for vacuolar degradation (Lu et al. 2025). Biochemical experiments have shown that OsER1 interacts directly with all six SINARs (Lu et al. 2025). In this study, we found that DEP2 exhibits a specific interaction with SINA3/5, while the other four SINA family members showed no interaction or only weak binding (Figure S1c). Grain width and weight were significantly increased in the *sina3‐cr*, *sina5‐cr* single mutants and *sina3 sina5* double mutant, whereas grain length showed a slight but non‐significant increase (Figure 4b). Previous studies have shown that overexpression of *DEP2*/*SUG1* has no significant effect on increasing the width of grains (Li et al. 2025). We speculated that deletion of *SINA3* and *SINA5*, which increases grain width, would disrupt the homeostasis of additional target proteins including OsER1, a recently characterized substrate of SINARs (Lu et al. 2025). Accordingly, other putative substrates of the SINA family are likely to participate in the modulation of grain size and grain weight. Moreover, in vitro ubiquitination and cell‐free protein degradation assays revealed that SINA3/5 mediate ubiquitination and degradation of DEP2 via the 26S proteasome pathway (Figure 2). These observations imply that DEP2 and OsER1 undergo distinct degradation pathways following ubiquitination by the SINA family.

In addition, SINA3 exhibits stronger ubiquitin ligase activity than SINA5, with higher levels of both auto‐ubiquitination and DEP2‐specific ubiquitination (Figure 2; Figure S6). Consistent with the robust ubiquitination activity of SINA3 toward DEP2, the *OE‐SINA3* overexpression lines showed a more pronounced reduction in grain size (Figure 5). SINA homologues contain an RING zinc finger domain and a SIAH‐type zinc finger domain, both of which are essential for ubiquitin ligase activity (Xia et al. 2020; Xie et al. 2002). The key amino acids in the RING domain of SINA3 and SINA5 are highly conserved, but there are six amino acid differences in the functional domain (Figure S2a). Amino acid substitution within the critical domains of E3 ubiquitin ligases is a key strategy for altering their enzymatic activity (Wang et al. 2023; Xia et al. 2020; Xie et al. 2002; Lu et al. 2025). For instance, substitution of a conserved cysteine (C) with serine (S), or a conserved histidine (H) with tyrosine (Y), in the RING domain reduces the ubiquitin ligase activity of SINATs (Xia et al. 2020). Notably, amino acid 148 in SINA5 is a tyrosine (Y), whereas SINA3 has a cysteine (C) at this position (Figure S2a). These amino acid differences may lead to variations in activity between SINA3 and SINA5; future research should focus on gaining a deeper understanding of the evolution and functional divergence of SINA proteins.

**FIGURE 6:**
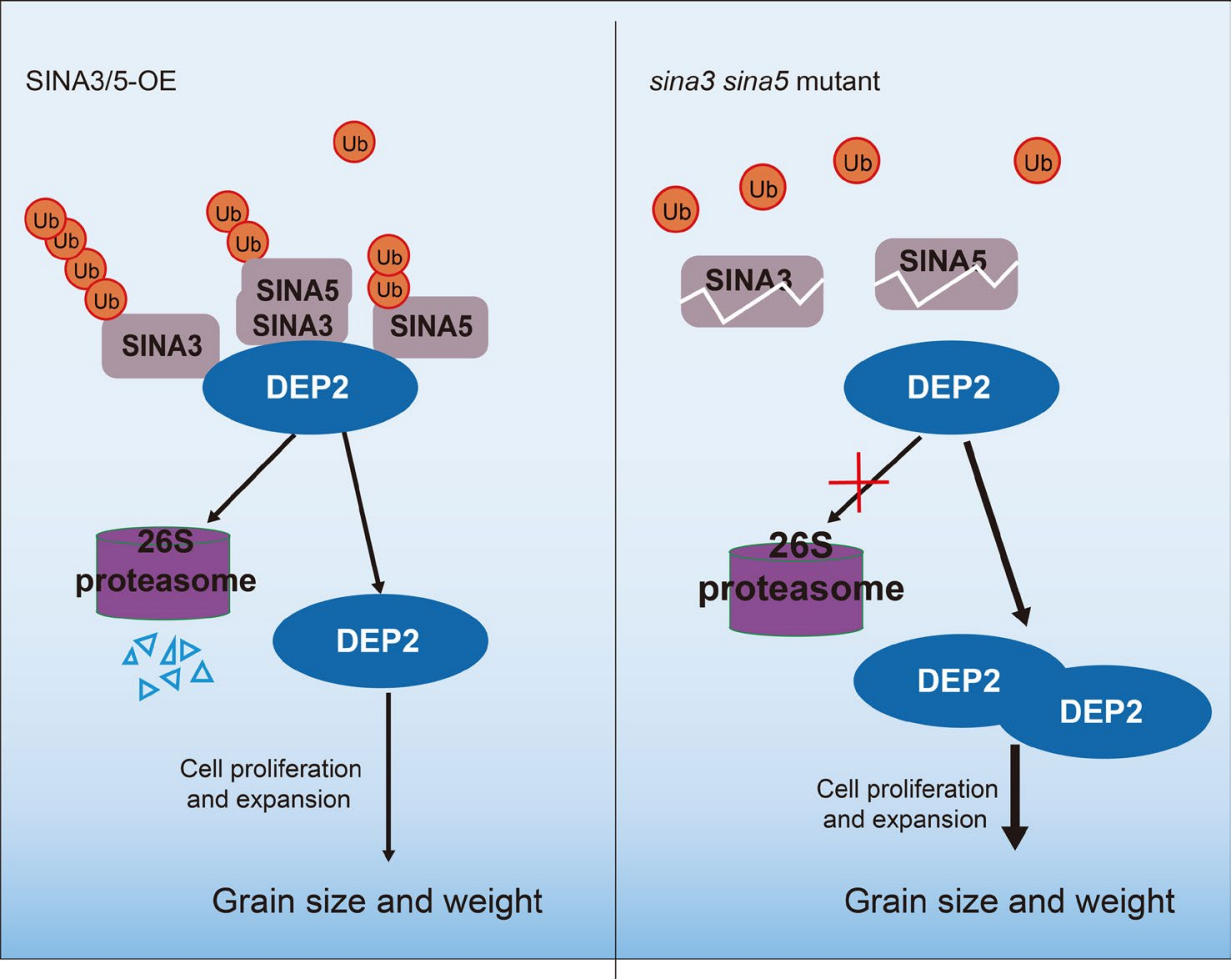
Working model for the roles of SINA3/5 in regulating grain size and weight by modulating DEP2 stability. The RING finger E3 ubiquitin ligases SINA3/5 interact with DEP2 to promote its ubiquitination and degradation via the 26S proteasome pathway. SINA3/5 regulate grain size and weight by modulating cell expansion and proliferation in the spikelet hull. Overexpression of *SINA3* and *SINA5* in the wild‐type background increases the degradation of DEP2, resulting in smaller grains and a decrease in grain weight. In the *sina3 sina5* double mutant, DEP2 protein exhibited retarded degradation and subsequent accumulation. This enhanced DEP2 functionality consequently promoted the expansion of spikelet cells, leading to increases in grain size and weight.

Based on the molecular and genetic evidence, we propose a working model to demonstrate how the SINA3/SINA5‐DEP2 regulatory module governs grain size and weight in rice (Figure 6). SINA3 and SINA5 negatively regulate grain size and weight by mediating DEP2 degradation, thereby coregulating cell expansion and proliferation in the spikelet hull. Nevertheless, the upstream molecular signals triggering SINA3/5 degradation remain poorly understood, representing a key direction for future investigation. Taken together, our findings reveal a critical SINA3/SINA5‐DEP2 regulatory module that governs grain size and weight in rice, representing a valuable target for improving crop grain yield through post‐translational regulation.

## 4 Experimental Procedures

### 4.1 Plant Materials and Growth Conditions

The *Oryza sativa* cv. Kitaake was used as the wild type and used for further transgenic analysis. All plants, including transgenic rice plants, were grown in the isolated paddy fields at the Chinese Academy of Agricultural Sciences in Beijing during the natural growing season or in Hainan during the winter season.

### 4.2 Phenotyping and Cellular Analysis

Mature grains were scanned using a ScanMarker i800 Plus scanner (MICROTEK, China). Grain length and width were measured with a vernier calliper. The lemma cell sizes and cell numbers in mature rice grains were observed using a SEM (SEM5000Pro, CIQTEK, China). ImageJ software was used to measure cell length and width. Cell numbers were determined based on the average grain length and cell length, as described by (Chen et al. 2024).

### 4.3 Vector Construction and Plant Transformation

To generate the *sina3‐cr* and *sina5‐cr* mutant, 20‐bp guide RNA (*SINA3*: TGTCAAATCTAATCCACGCG, *SINA5*: TACTACGCAAACTCAAAGCA) was designed and the expression cassette was inserted into CRISPR/Cas9 expression construct. To obtain *sina3 sina5* double mutant, two guide RNA fragments were cloned and inserted into the sgRNA‐Cas9 vector pHUE411, which is a CRISPR/Cas9 toolkit for multiplex genome editing (Xing et al. 2014). To produce the *OE‐SINA3* and *OE‐SINA5* rice plants, the full‐length coding sequence (CDS) of wild‐type *SINA3* and *SINA5* was cloned and inserted into the binary vector pCAMBIA2300 to generate the construct *2300‐proUbi:SINA3* and *2300‐proUbi:SINA5*. These constructs were introduced into Kitaake calli via an *Agrobacterium*‐mediated transformation. The primers used for vector construction and plant transformation are listed in Table S3.

### 4.4 RNA Extraction and RT‐qPCR Analysis

Various tissues at the flowering stage were collected from Kitaake, *dep2‐3* and transgenic lines. Total RNA was extracted using an RNAprep Pure Plant Kit (Tiangen Co., Beijing, China). About 1 μg of total RNA was used for first‐strand cDNA synthesis in a 20 μL volume with PrimeScript II Reverse Transcriptase (TaKaRa, Dalian, China). Real‐time PCR was performed on an ABI 7500 real‐time PCR system with the SYBR Premix Ex Taq Kit (Takara, Shiga, Japan). Rice *UBIQUITIN* (*LOC_Os03g13170*) gene was used as the internal control. The primers used for analysis are listed in Table S3.

### 4.5 Subcellular Localization Assay

To investigate the subcellular localization of SINA3 and SINA5 in *N. benthamiana* leaves, the full‐length of SINA3/5 proteins was fused with GFP in the pCAMBIA1305 plasmid using *XbaI* and *BamHI* and expressed via the 35S promoter. The full‐length of DEP2 protein was fused with GFP or mCherry in the pCAMBIA1305 plasmid using *XbaI* and *BamHI* and expressed via the 35S promoter. These constructs and markers were introduced into *Agrobacterium* strain EHA105 and co‐infiltrated into *N. benthamiana* leaves for subcellular localization analysis (Waadt and Kudla 2008). Images were captured using a laser scanning confocal microscope (LSM980; Zeiss, Baden‐Wurttemberg, Germany).

For subcellular localization of SINA3 and SINA5 in rice protoplasts, the full‐length CDS of *SINA3* and *SINA5* were cloned and inserted into the pAN580‐GFP vector (at the *XbaI* and *BamHI* sites), generating the fusion constructs *pAN580‐SINA3‐GFP* and *pAN580‐SINA5‐GFP*. The method for preparing rice protoplasts is referenced in a previous report (Zhang et al. 2011). Confocal imaging was performed using a Zeiss LSM980 laser scanning confocal microscope.

### 4.6 Yeast Two‐Hybrid Assay

A yeast two‐hybrid assay was used to detect protein interactions using the Matchmaker Two‐Hybrid System (Clontech, CA, USA). The coding region of the full‐length DEP2 protein (DEP2^FL^, amino acids 1–1365), the N‐terminal domain (DEP2^NT^, amino acids 1–691), the C‐terminal domain (DEP2^CT^, amino acids 690–1365), the C1 domain (DEP2^C1^, amino acids 690–1024) and the C2 domain (DEP2^C2^, amino acids 1023–1365) were cloned into the *EcoRI* and *BamHI* restriction sites of the pGBKT7 vector (bait), and the coding region of OsSINA1‐5 and OsDIS1 were separately cloned into the pGADT7 vector (prey). Various combinations of plasmids were cotransformed into the yeast strain AH109 (Clontech). After growing on SD‐Trp/−Leu plates for 3 days at 30°C, interactions were observed on the selective medium SD‐Leu/−Trp/−His/−Ade. Representative results were obtained from three biological replicates. Primers used for this assay are listed in Table S3.

### 4.7 Bimolecular Fluorescence Complementation (BiFC) Assays

The full‐length CDS of *SINA3* and *SINA5* were ligated into the N‐terminal fragment of YFP in pSPYNE vector to generate the eYNE‐SINA3 and eYNE‐SINA5 constructs, and the full‐length CDS of DEP2 was ligated into the C‐terminal fragment of YFP in pSPYCE vector to generate the DEP2‐eYCE construct. These constructs were introduced into *Agrobacterium* strain EHA105, and various combinations of EHA105 strains were co‐infiltrated into *N. benthamiana* leaves as previously described (Waadt and Kudla 2008). The leaf epidermal cells of *N. benthamiana* were transfected for 48 h and treated with 50 μM MG132. Confocal imaging was performed using a laser scanning confocal microscope (LSM980; Zeiss, Baden‐Wurttemberg, Germany). Representative results were obtained from three biological replicates. Primers used for this assay are listed in Table S3.

### 4.8 In Vivo Co‐Immunoprecipitation Assay

The C1 domain (DEP2^C1^, amino acids 690–1024) of *DEP2* was cloned and inserted into the pCAMBIA1300‐Flag vector. Similarly, the full‐length CDSs of *SINA3* and *SINA5* were amplified and ligated into the pCAMBIA1305‐GFP vector at the XbaI and BamHI restriction sites, generating the respective fusion constructs. The constructed vectors were transformed into EHA105 and used to infect *N. benthamiana* leaves in the presence or absence of 50 μM MG132. After co‐expression for 2 days, total protein was extracted from freshly harvested leaves at a sample‐to‐buffer ratio of 100 mg: 300 μL (w/v). in a NB1 buffer (50 mM Tris‐MES, pH 8.0, 1 mM MgCl_2_, 0.5 M sucrose, 10 mM EDTA, 5 mM DTT and 1× proteinase inhibitor cocktail). Subsequently, the protein extracts were incubated with Anti‐GFP beads (D153‐11; Medical & Biological Laboratories (MBL), Tokyo, Japan) at 4°C for 2 h. Beads were rinsed three times with the NB1 buffer and boiled in 1× SDS‐PAGE sample buffer for immunoblot analysis. Anti‐Flag (M185‐7; MBL, Tokyo, Japan) or anti‐GFP (598–7; MBL, Tokyo, Japan) antibodies were used at a dilution of 1:2000.

### 4.9 Protein Expression and Purification

To generate GST‐ and His‐tag fusion proteins, the CDSs of SINA3, SINA5 and DEP2 were amplified and cloned into the vectors pGEX4T‐1 or pET30a. The GST‐SINA3, GST‐SINA5, His‐DEP2‐Flag and truncated His‐DEP2^C1^ proteins were expressed in cells of *Escherichia coli* strain BL21 (DE3) (TransGen, Beijing, China) under induction with 0.5 mM isopropyl‐b‐D‐thiogalactoside (IPTG) while shaking at 16°C for 16 h. The fusion proteins were purified using Beaver Beads GSH (70601–100; Beaver, Suzhou, China) and IDA‐Nickel (70501‐K100; Beaver, Suzhou, China), respectively.

### 4.10 Pull‐Down Assay

To investigate the DEP2‐SINA3/5 interaction with an in vitro glutathione s‐transferase (GST) pull‐down assay, equal amounts of GST‐SINA3, GST‐SINA5 or GST proteins adsorption on beads were mixed with His‐DEP2‐Flag or His‐DEP2^C1^, followed by incubation for 2 h at 4°C. The anti‐GST beads (70601–100; Beaver Beads GSH, Suzhou, China) were washed three times with washing buffer (25 mmol/L Tris–HCl, 50 mmol/L NaCl, 1 mmol/L DTT, 1% Triton X‐100 and 0.1% SDS) and boiled with 100 μL 1× protein loading buffer at 95°C for 10 min. The proteins were separated in 10% SDS‐PAGE gels and detected by immunoblotting using anti‐GST antibodies (PM013‐7; MBL, 1:5000) and anti‐His antibodies (D291‐7; MBL, 1:5000).

### 4.11 In Vitro Ubiquitination Assay

In vitro ubiquitination assay was performed as described previously (Wang et al. 2024). Recombinant His‐DEP2‐Flag, His‐DEP2^C1^, GST‐SINA3, GST‐SINA5, E1 (His‐AtUBA2) and E2 (His‐AtUBC10) were purified using Beaver Beads GSH (70601‐100; Beaver, Suzhou, China) and IDA‐Nickel (70501‐K100; Beaver, Suzhou, China), respectively. A total of 50 ng E1, 100 ng E2, 2 μg Flag‐Ub (U120; BostonBiochem, Massachusetts, USA), 2 μg GST‐SINA3 or GST‐SINA5 and 2 μg His‐DEP2‐Flag or His‐DEP2^C1^ were incubated in ubiquitination reaction buffer (50 mM Tris–HCl pH 7.5, 10 mM MgCl_2_, 5 mM ATP and 2 mM DTT) at 30°C with incubation for 1 h. After reaction, the samples were added to 5× protein loading buffer and then boiled for 5 min at 95°C, which were subjected to immunoblotting with anti‐GST and anti‐His antibodies (MBL, PM013‐7 and D291‐7, 1:5000 dilution). The polyubiquitination of DEP2 was detected with anti‐ubiquitin (P4D1; Cell Signalling Technology, 1:1000 dilution), anti‐K48‐linked ubiquitin (A18163; ABclonal, 1:1000 dilution), anti‐K63‐linked ubiquitin (A18164; ABclonal, 1:1000 dilution) and anti‐Flag (M185‐7; MBL, 1:5000 dilution) antibodies.

### 4.12 Cell‐Free Protein Degradation Assay In Vitro

The fusion proteins of His‐DEP2‐Flag and His‐DEP2^C1^ were obtained by the IPTG induction system. Cell‐free protein degradation was assayed as described previously (Lin et al. 2012). In brief, total proteins were extracted from rice seedlings with the extraction buffer (25 mM Tris‐MES, pH 7.5, 10 mM MgCl_2_, 10 mM NaCl, 100 mM PMSF, 5 mM DTT). Equal amounts of extracts were incubated with or without 100 μM MG132. Samples were taken at indicated intervals for the western blotting using the anti‐His (PM013‐7; MBL, 1:5000 dilution), anti‐Flag (M185‐7; MBL, 1:5000 dilution) and anti‐HSP82 (AbM51099‐31‐PU; BPI, 1:5000 dilution) antibodies.

### 4.13 LC–MS Analysis

LC–MS analysis was performed as the method described previously (van der Wal et al. 2018). To perform liquid chromatography‐mass spectrometry (LC–MS), we first performed ubiquitination on the purified full‐length DEP2 and truncated His‐DEP2^C1^ proteins in vitro. The reactions were performed with 50 ng E1, 100 ng E2, 2 μg Flag‐Ub (U120; BostonBiochem, Massachusetts, USA), 2 μg GST‐SINA3 and 2 μg His‐DEP2‐Flag or His‐DEP2^C1^ were incubated in ubiquitination reaction buffer (50 mM Tris–HCl pH 7.5, 10 mM MgCl_2_, 5 mM ATP and 2 mM DTT) at 30°C with incubation for 1 h. The reaction products were separated on 10% SDS‐PAGE gels and stained with coomassie brilliant blue. Gel bands corresponding to ubiquitinated His‐DEP2‐Flag and His‐DEP2^C1^ proteins were excised and subjected to LC–MS analysis. The bands representing ubiquitinated His‐DEP2‐Flag and His‐DEP2^C1^ were excised for LC–MS analysis. The LC–MS assay was performed by Beijing Protein Innovation (http://www.proteomics.org.cn/). NanoLC–MS mass spectra were acquired on an Oribtrap Tribrid Lumos mass spectrometer (Thermo) coupled to an EASY‐nLC 1200 system (Thermo Fisher Scientific). Peptides were separated on an in‐house packed 150 mM inner diameter column containing 15 cm Reprosil‐pur 120 C18‐AQ phase resin (1.9 mm, 120 A°, Dr. Maisch, Germany) with a gradient consisting of 2%–35% (80% AcN, 0.1% FA) over 120 min at 300 nL/min. MS1 spectra were collected at a resolution of 120 000 with an automated gain control (AGC) target of 4E5 and a max injection time of 50 ms. Precursors were filtered according to charge state (2–5z), and monoisotopic peak assignment. Previously interrogated precursors were dynamically excluded for 60 s. Peptide precursors were isolated with a quadrupolemass filter set to a width of 1.6 Th. Ion trap MS2 spectra were collected at an AGC of 1E4, maximum injection time of 50 ms and HCD collision energy of 32%. For data analysis, Lysine with a diGly remnant and oxidation of methionine were set as variable modifications. Carbamidomethylation of cysteine was set as a fixed modification. The false discovery rate was set to 1% and the minimum score for diGly peptides was set to 20.

### 4.14 Protein Extraction and Immunoblot Analysis

Polyclonal antibody against DEP2 was raised in rabbit with purified a DEP2 middle region protein (529–726 aa) at ABclonal Technology Co. (https://www.abclonal.com.cn/). CDS (1587–2178) of DEP2 was inserted into pET‐28a‐SUMO vector and the protein was purified with Ni–NTA agarose. The specificities of anti‐DEP2 polyclonal antibodies were determined by immunoblot analysis using wild‐type and *dep2‐3* young panicle tissues, together with purified full‐length His‐DEP2‐Flag protein. The fresh young panicles were taken to extract protein using NB1 buffer, then incubated at 4°C for 30 min with rotation, followed by centrifugation at 12 000*g* for 15 min at 4°C. The extracted total proteins were boiled at 95°C for 5 min with 1× protein loading buffer. The extracted supernatant proteins were separated by SDS‐PAGE (10% gels). Proteins were then transferred to the NC membrane, detected with anti‐DEP2 antibodies at 1:2000 dilutions and visualized with enhanced chemiluminescence reagent (GE Healthcare). Anti‐HSP82 antibodies (AbM51099‐31‐PU; Beijing Protein Innovation) were used as the loading controls.

### 4.15 Statistical Analysis

Data are shown as mean ± SD (standard deviation), calculated using GraphPad Prism 8.02 version. One‐way analysis of variance (ANOVA) followed by Tukey's tests was used for pairwise multiple comparisons. Student's *t*‐tests were used for significant difference analysis between two samples. *p*‐value < 0.05 was considered statistically significant. Statistical analysis and number of biologically independent samples (*n*) are indicated in the figure legends. Images were analysed with ImageJ.

### 4.16 Accession Numbers

Sequence data for this article can be found in the Rice Annotation Project Database (RAP‐DB) and GenBank database under the following accession numbers: *DEP2*, *LOC_Os07g42410*; *SINA1*, *LOC_Os07g46560*; *SINA2*, *LOC_Os02g03620*; *SINA3*, *LOC_Os05g14860*; *SINA4*, *LOC_Os01g13370*; *SINA5*, *LOC_Os02g19140*; *OsDIS1*, *LOC_Os03g24040*; *UBIQUITIN*, *LOC_Os03g13170*; *AtUBA2*, *AT5G06460*; and *AtUBC10*, *At5g53300*.

## Author Contributions

J.W. and L.J. supervised the study. H.W., X.S. and X.W. conceived the study and designed the experiments. C.X., Y.L., X.Y., Y.Z., X.H., Y.Z., Y.L. and Y.X. participated in partial biochemical experiments. Q.L., C.L., X.G., S.Z., Z.C., Y.R. and L.J. participated in material development. H.W. drafted the manuscript, and L.J. and X.W. revised the manuscript. All authors contributed to subsequent versions and have read and approved the manuscript.

## Funding

This work was supported by the National Key Research and Development Program of China (2021YFD1200504, 2021YFF1000200), Biological Breeding‐National Science and Technology Major Project (2023ZD0406902), the Special Modern Agricultural Foundation of Jiangsu Key R&D project (BE2023362).

## Conflicts of Interest

The authors declare no conflicts of interest.

## Supporting information


**Figure S1.** Screening and identification of DEP2‐interacting proteins.
**Figure S2.** Multiple amino acid sequence alignment and dimerization analyses of six SINA proteins in rice.
**Figure S3.** Subcellular localization of SINA3 and SINA5 in rice protoplasts.
**Figure S4.** Co‐expression of SINA3 and SINA5 with DEP2 in the leaf epidermal cells of *N. benthamiana*.
**Figure S5.** Purified recombinant proteins for biochemical studies.
**Figure S6.** SINA3 and SINA5 are E3 ubiquitin ligases with self‐ubiquitination activity in vitro.
**Figure S7.** Ubiquitination of DEP2 and DEP2^C1^ by GST‐SINA3 or GST‐SINA5 in vitro.
**Figure S8.** Liquid chromatography‐mass spectrometry (LC–MS) spectra of ubiquitinated peptides from DEP2.
**Figure S9.** Knockout of *SINA3* and *SINA5* in the Kitaake background.
**Figure S10.** Plant morphology of wild‐type, *sina3‐cr*, *sina5‐cr* and *sina3 sina5* transgenic lines.
**Figure S11.** Antibody‐specific detection of DEP2 antibody.
**Figure S12.** Expression levels of *SINAs* genes in different genetic materials.
**Figure S13.** Expression levels of *DEP2* and *SINA3/5* genes in different tissues.
**Figure S14.** Overexpression of *SINA5* in the *dep2‐3* mutant background.
**Table S1.** Agronomic traits of WT, *sina3‐cr*, *sina5‐cr* and *sina3 sina5* transgenic lines.
**Table S2.** Agronomic traits of WT, *dep2‐3*, *OE‐SINA3* (−1, −2 and −3) and *OE‐SINA5* (1, −2 and −3) transgenic lines.
**Table S3:** Primers used in this work.

## Data Availability

The data that support the findings of this study are available on request from the corresponding author. The data are not publicly available due to privacy or ethical restrictions.
