# Supplementary material for: The E3 Ubiquitin Ligases SINA3 and SINA5 Control DEP2 Ubiquitination and Proteasomal Degradation to Regulate Grain Size and Weight in Rice
Source: Plant Biotechnol J. 2026 Jul 17;24(10):5483–97. doi: 10.1111/pbi.70723 (PMC13398880; doi:10.1111/pbi.70723)
Supplement: Supplementary file 1 [file PBI-24-5483-s001.docx]

**Supporting Information for “The E3 ubiquitin ligases SINA3 and SINA5 control DEP2 ubiquitination and proteasomal degradation to regulate grain size and weight in rice”**

**Figure S1 Screening and identification of DEP2-interacting proteins.** (a) Using BD-DEP2 as the bait protein to screen DEP2-interacting proteins from yeast library and 11 potential interacting proteins with functional annotations were selected. (b) Y2H assay confirming that DEP2 interacts with full-length OsSINA5 annotated as a putative E3 ubiquitin ligase. (c) DEP2 also interacts with OsSINA3, the homologs of OsSINA5. Transformed yeast cells were spotted on the control medium DDO (SD/-Trp/-Leu) and selective medium QDO (SD/-Trp/-Leu/-His/-Ade).

**Figure S2 Multiple amino acid sequence alignment and dimerization analyses of six SINA proteins in rice.** (a) Multiple sequence alignment of six SINA family proteins using ClustalX and the graphic was drawn with GeneDoc. The purple lines represent RING-type zinc finger domain and red lines represent SIAH-type zinc finger domain. Purple circles and red triangles indicate conserved cystine and histidine residues within the RING finger and ZINC finger, respectively. The red box with numbers 1–6 below the sequence indicate the amino acid differences between OsSINA3 and OsSINA5 within the zinc finger domain. (b) Domain structures of six OsSINA proteins. RING, RING finger; SINA-type domain, SINA-type domain contains ZINC finger. The C-terminal has a conserved SINA domain, which is involved in substrate binding and dimerization. (c) Pairwise yeast two-hybrid interaction of OsSINA proteins. OsSINA proteins can interact with each other, forming homo- or hetero-dimers. Transformed yeast cells were spotted on the selective medium QDO (SD/-Trp/-Leu/-His/-Ade).

**Figure S3 Subcellular localization of SINA3 and SINA5 in rice protoplasts.** (a–c) Subcellular localization of SINA3-GFP and SINA5-GFP in rice protoplasts. D53-mCherry was used as a nucleus marker. Bars = 5 *µ*m.

**Figure S4 Co-expression of SINA3 and SINA5 with DEP2 in the leaf epidermal cells of *N. benthamiana*.** (a–c) Transient expression DEP2-mcherry, SINA3-GFP and SINA5-GFP in the leaf epidermal cells of *N. benthamiana,* the fluorescence signals are dispersed in the cytoplasm and nuclear. Co-expression of SINA3-GFP or SINA5-GFP and DEP2-mCherry in the leaf epidermal cells of *N. benthamiana* treated with 50 *µ*M MG132. Bars = 10 *µ*m.

**Figure S5 Purified recombinant proteins for biochemical studies.** (a) Purified proteins for Pull-down and *in vitro* ubiquitination assays. Coomassie blue stained SDS-PAGE gel of purified His-DEP2-Flag and protein His-DEP2^C1^. (b) Protein purification for ubiquitination experiment *in vitro*. Coomassie blue stained SDS-PAGE gel of purified His-E1 (AtUBA1), His-E2 (AtUBC10), GST-SINA3 and GST-SINA5. BSA was used as a standard.

**Figure S6 SINA3 and SINA5 are E3 ubiquitin ligases with self-ubiquitination activity *in vitro*.** We observed self-ubiquitination of GST–SINA3 and GST–SINA5 *in vitro*. Polyubiquitination was detected by immunoblotting with anti-Ub antibodies (1:1,000 dilution) and anti-His antibodies (1:5,000 dilution). “**+**” and “**–**” indicate the presence and absence of the components in each reaction mixture, respectively.

**Figure S7 Ubiquitination of DEP2 and DEP2^C1^ by GST-SINA3 or GST-SINA5 *in vitro*.** (a, b) The polyubiquitination was detected by immunoblotting with anti-K63Ub and anti-His antibodies. “**+**” and “**–**” indicate the presence and absence of the components in each reaction mixture, respectively.

**Figure S8 Liquid chromatography-mass spectrometry (LC-MS) spectra of ubiquitinated DEP2 peptides by SINA3**. (a) K399 in the DEP2 peptide EIGTPVGTPTSANLEVNS**K**AR was ubiquitinated. The theoretical MW is 2254.1599, z is 3, and m/z = 752.3939. (b) K722 in the DEP2 peptide LFAAQ**K**LTSSR was ubiquitinated. The theoretical MW is 1334.7392, z is 2, and m/z = 668.3769. (c) K746 in the DEP2 peptide STDVQVENTPRVNEV**K**PPLVLPER was ubiquitinated. The theoretical MW is 2829.5011, z is 3, and m/z = 944.1743. (d) K962 in the DEP2 peptide NLPDNPLAQSVPNFADLRKENT**K**PSAGLSR was ubiquitinated. The theoretical MW is 3362.7267, z is 5, and m/z = 673.5526. (e) K1344 in the DEP2 peptide LREDQLTGSSL**K**APR was ubiquitinated. The theoretical MW is 1783.9605, z is 3, and m/z = 595.6608. Two glycines from ubiquitin were found on lysine in these peptides.

**Figure S9 Knockout of *SINA3* and *SINA5* in the Kitaake background.** (a, b) Schematic diagrams of the CRISPR target fragment (in green) in the exon of *SINA3* and *SINA5.* The position of the protospacer adjacent motif (PAM) sequence is underlined (yellow). Insertions or deletions of bases are marked in red. Boxes and lines indicate exons and introns, respectively. The *sina3 sina5* double mutant was generated via double knockout vectors.

**Figure S10 Plant morphology of wild-type, *sina3-cr*, *sina5-cr*, and *sina3 sina5* transgenic lines.** Plant and panicle morphology of wild-type (Kitaake) and mutant alleles at the reproductive stages. Bar = 10 cm.

**Figure S11 Antibody-specific detection of DEP2 antibody****.** (a, b) DEP2 antibodies can specifically detect endogenous DEP2 protein in young panicle tissues of wild-type and purified full-length His-DEP2-Flag protein but not in the *dep2-3* mutant (a). Pre-immune serum (b) was used as a control. Molecular weights of proteins (kDa) are shown on the left. The arrowhead indicates the DEP2 protein in wild-type young panicles and purified His-DEP2-Flag protein.

**Figure S12 Expression levels of *SINAs* genes in different genetic materials.** (a, b) RT-qPCR analyses of *SINA3* (a) and *SINA5* (b) expression in wild-type (WT), *sina3-cr*, *sina5-cr*, and *sina3 sina5* transgenic lines. (c) RT-qPCR analyses of *SINA1*, *SINA2*, *SINA4*, and *OsDIS* expression in WT, *sina3-cr*, *sina5-cr*, and *sina3 sina5* transgenic lines. Rice *UBIQUITIN* gene was used as the internal control. Representative results from three biological replicates were shown. Values are means ± SD (*n* = 3). Asterisks indicate significant differences compared with the WT, according to Student’s *t*-test (***P* < 0.01).

**Figure S13 Expression levels of *DEP2* and *SINA3/5* genes in different tissues.** (a) RT-qPCR analyses of *DEP2* expression in various tissues, including young spikelet at different developmental stages. (b, c) RT-qPCR analyses of *SINA3* and *SINA5* expression in various tissues. Rice *UBIQUITIN* gene was used as the internal control. Representative results from three biological replicates were shown. Values are means ± SD (*n* = 3). The red line shows the dynamic trend of gene expression levels.

**Figure S14 Overexpression of *SINA5* in the *dep2-3* mutant background.** (a) Grain comparisons of WT, *dep2-3*, and *2300-proUbi:SINA5* transgenic lines. #1, #2 and #3 represent three independent overexpression lines. Bar = 1 cm. (b) Relative expression levels of *SINA5* in WT, *dep2-3*, and *2300-proUbi:SINA5* transgenic lines. Rice *UBIQUITIN* gene was used as an internal control. Values are means ± SD (*n* = 3). Asterisks indicate significant differences compared with the WT, according to Student’s *t*-test (***P* < 0.01). (c–e) Comparisons of grain length (c), grain width (d) and 1,000-grain weight (e) of WT, *dep2-3*, and *2300-proUbi:SINA5* transgenic lines. Values are means ± SD (*n* ≥ 18 in c, d and *n* ≥ 3 in e). Different letters indicate significant differences ranked by pairwise multiple comparison followed by Tukey’s test (*P* < 0.05).

**Table S1 Agronomic traits of WT, *sina3-cr*, *sina5-cr*, and *sina3 sina5* transgenic lines.**

| Agronomic traits | WT | *sina3-cr-1* | *sina3-cr-2* | *sina5-cr-1* | *sina5-cr-2* | *sina3 sina5* |
| --- | --- | --- | --- | --- | --- | --- |
| Plant height (cm) | 67.33 ± 2.24 | 68.58 ± 3.12 | 67.71 ± 2.02 | 68.37 ± 3.61 | 66.00 ± 2.19 | 65.67 ± 2.90 |
| Tiller number | 14.5 ± 1.56 | 14.25 ± 1.54 | 13.75 ± 1.66 | 15.50 ± 1.56 | 15.66 ± 1.56 | 14.33 ± 1.49 |
| Panicle length (cm) | 12.47 ± 0.58 | 12.92 ± 0.76 | 12.91 ± 0.75 | 12.83 ± 0.81 | 13.00 ± 0.90 | 12.25 ± 0.65 |
| Grain number per panicle | 51.33 ± 6.71 | 54.61 ± 7.52 | 49.83 ± 7.71 | 54.17 ± 6.13 | 52.91 ± 6.40 | 51.33 ± 5.16 |
| Grain thickness (mm) | 2.43 ± 0.06 | 2.40 ± 0.09 | 2.39 ± 0.09 | 2.39 ± 0.06 | 2.36 ± 0.05 | 2.39 ± 0.05 |

Values are means ± SD (*n* = 12). Student’s *t*-test.

**Table S2** **Agronomic traits of WT, *dep2-3*, *OE-SINA3* (-1, -2 and -3), and *OE-SINA5* (1, -2 and -3) transgenic lines.**

| Agronomic traits | WT | *dep2-3* | *OE-SINA3-1* | *OE-SINA3-2* | *OE-SINA3-3* | *OE-SINA5-1* | *OE-SINA5-2* | *OE-SINA5-3* |
| --- | --- | --- | --- | --- | --- | --- | --- | --- |
| Plant height (cm) | 67.64 ± 2.07 | 50.92 ± 2.17** | 49.87 ± 2.75** | 53.41 ± 1.95** | 54.04 ± 2.82** | 52.79 ± 4.41** | 54.54 ± 3.25** | 54.71 ± 4.33** |
| Tiller number | 15.25 ± 1.28 | 11.67 ± 1.56** | 15.33 ± 1.97 | 14.33 ± 1.55 | 17.33 ± 1.30* | 15.00 ± 1.75 | 16.25 ± 1.14 | 16.42 ± 2.57 |
| Panicle length (cm) | 12.66 ± 0.94 | 8.25 ± 0.50** | 10.25 ± 0.69** | 10.62 ± 0.43** | 11.62 ± 0.77** | 11.25 ± 0.54** | 10.25 ± 0.78** | 11.83 ± 0.44* |
| Grain number per panicle | 49.58 ± 8.26 | 46.66 ± 11.46* | 45.83 ± 4.24* | 48.66 ± 5.72 | 45.50 ± 4.91* | 52.42 ± 6.37 | 48.42 ± 7.60 | 46.75 ± 3.49 |
| Grain thickness (mm) | 2.41 ± 0.04 | 2.48 ± 0.05* | 2.28 ± 0.07** | 2.36 ± 0.06 | 2.36 ± 0.07 | 2.35 ± 0.11 | 2.37 ± 0.07 | 2.37 ± 0.06 |

Values are means ± SD (*n* = 12). Student’s *t*-test (**P* < 0.05; ***P* < 0.01).

**Table S3 Primers used in this work.**

| **Purpose** | **Primer name** | **Primer sequence (5’ to 3’)** |
| --- | --- | --- |
| Constructs for overexpression | 2300ubi-SINA3-F | GTAGAAGAGGTACCCGGGATGGACATGGACAGCGTGGAG |
|  | 2300ubi-SINA3-R | CTCTAGAGGATCCCCGGGTCAGCTACAAAGGTTCGGTATG |
|  | 2300ubi-SINA5-F | GTAGAAGAGGTACCCGGGATGGACATGGCGAGCATCGA |
|  | 2300ubi-SINA5-R | CTCTAGAGGATCCCCGGGGCTGAATAGGTTTGGAATGC |
| Constructs for gene knockout | CRISPR-SINA3-F | AGATGATCCGTGGCATGTCAAATCTAATCCACGCGGTTTTAGAGCTATGC |
|  | CRISPR-SINA3-R | GCATAGCTCTAAAACCGCGTGGATTAGATTTGACATGCCACGGATCATCT |
|  | CRISPR-SINA5-F | AGATGATCCGTGGCATGCTTGAGTTTGCTGTAGTAGTTTTAGAGCTATGC |
|  | CRISPR-SINA5-R | GCATAGCTCTAAAACTACTACAGCAAACTCAAGCATGCCACGGATCATCT |
|  | pHUE411-SINA3/5-F | TTTGTAGGTAGACAAAGCTTGTCGAGGCTGAGTAAGGTTAACTT |
|  | pHUE411-SINA3/5-R | AAGTTAACCTTACTCAGCCTCGACAAGCTTTGTCTACCTACAAA |
| Constructs for prokaryotic expression | pET30a-DEP2-Flag-F | GGCTGATATCGGATCCATGGAGCCCGACGCCCCGCTC |
|  | pET30a-DEP2-Flag-R | GACGGAGCTCGAATTCCCTGAGCCTTGCATCACC |
|  | pET30a-DEP2^C1^-F | GGCTGATATCGGATCCTTGGAGCAGACTAAGAGACCGAC |
|  | pET30a-DEP2^C1^-R | GACGGAGCTCGAATTCGTATATGACGTCATTCATGGATG |
|  | pET30a-AtE1-F | AGGAGATATACATATGATGGAACCATTCGTTGTTAAGG |
|  | pET30a-AtE1-R | TGATGATGGTGCATATGGGCGAAGTAGACTGATACG |
|  | pET30a-AtE2-F | AGGAGATATACATATGATGGCGTCGAAGCGGAT |
|  | pET30a-AtE2-R | TGATGATGGTGCATGCCCATGGCATACTTCTGAGT |
|  | pGEX-SINA3-F | GGTTCCGCGTGGATCCATGGACATGGACAGCGTGGAGT |
|  | pGEX-SINA3-R | GTCGACCCGGGAATTCTCAGCTACAAAGGTTCGGTATGC |
|  | pGEX-SINA5-F | GGTTCCGCGTGGATCCATGGACATGGCGAGCATCG |
|  | pGEX-SINA5-R | GTCGACCCGGGAATTCTCAGCTGAATAGGTTTGGAA |
| Constructs for protein localization | pAN580-SINA3-GFP-F | CGGAGCTAGCTCTAGAATGGACATGGACAGCGTGGAGTGC |
|  | pAN580-SINA3-GFP-R | TGCTCACCATGGATCCGCTACAAAGGTTCGGTATGC |
|  | pAN580-SINA5-GFP-F | CGGAGCTAGCTCTAGAATGGACATGGCGAGCATCGA |
|  | pAN580-SINA5-GFP-R | TGCTCACCATGGATCCGCTCAGTTGAATAGGTTTGG |
|  | 1305-DEP2-mCherry-F | CGGAGCTAGCTCTAGAATGGAGCCCGACGCCCCGCTC |
|  | 1305-DEP2-mCherry-R | TGCTCACCATGGATCCCCTGAGCCTTGCATCACCCC |
| Constructs for CoIP assays | 1305-SINA3-GFP-F | CGGAGCTAGCTCTAGAATGGACATGGACAGCGTGGAGTGC |
|  | 1305-SINA3-GFP-R | TGCTCACCATGGATCCGCTACAAAGGTTCGGTATGC |
|  | 1305-SINA5-GFP-F | CGGAGCTAGCTCTAGAATGGACATGGCGAGCATCGA |
|  | 1305-SINA5-GFP-R | TGCTCACCATGGATCCGCTCAGTTGAATAGGTTTGG |
|  | 1300-Flag-DEP2-F | ATGATAAGGGCGGTACCATGGAGCCCGACGCCCCGCTCGAC |
|  | 1300-Flag-DEP2-R | AGGCTACGTAGGATCCTCACCTGAGCCTTGCATCACCC |
|  | 1300-Flag-DEP2^C1^-F | ATGATAAGGGCGGTACCTTGGAGCAGACTAAGAGACCGAC |
|  | 1300-Flag-DEP2^C1^-R | AGGCTACGTAGGATCCTCAATTGTATATGACGTCATTCAT |
| Constructs for yeast two-hybrid assays | BD-DEP2-F | CATGGAGGCCGAATTCATGGAGCCCGACGCCCCGCTCG |
|  | BD-DEP2-R | GCAGGTCGACGGATCCTCACCTGAGCCTTGCATCACC |
|  | BD-DEP2^NT^-F | CATGGAGGCCGAATTCATGGAGCCCGACGCCCCGCTCG |
| Constructs for yeast two-hybrid assays | BD-DEP2^NT^-R | GCAGGTCGACGGATCCTCACTCCAAAGGAACAGCATTTCC |
|  | BD-DEP2^CT^-F | CATGGAGGCCGAATTCTTGGAGCAGACTAAGAGACCGAC |
|  | BD-DEP2^CT^-R | GCAGGTCGACGGATCCTCACCTGAGCCTTGCATCACC |
|  | BD-DEP2^C1^-F | CATGGAGGCCGAATTCTTGGAGCAGACTAAGAGACCGAC |
|  | BD-DEP2^C1^-R | GCAGGTCGACGGATCCTCAATTGTATATGACGTCATTCAT |
|  | BD-DEP2^C2^-F | CATGGAGGCCGAATTCTACAATTGGGCTCCCTCAAAAAT |
|  | BD-DEP2^C2^-R | GCAGGTCGACGGATCCTCACCTGAGCCTTGCATCACC |
|  | AD-COP1-F | GGAGGCCAGTGAATTCATGGGTGACTCGACGGTGGCCGGC |
|  | AD-COP1-R | CGAGCTCGATGGATCCTCAAGGAGCAAGTACAAGAAC |
|  | AD-SINA1-F | GGAGGCCAGTGAATTCATGGCATCAGTTACTTATCTTG |
|  | AD-SINA1-R | CGAGCTCGATGGATCCTCACTGCTCCTTCCAAATCCTTC |
|  | AD-SINA2-F | GGAGGCCAGTGAATTCATGGCCCCAGGAAGCAGCATTG |
|  | AD-SINA2-R | CGAGCTCGATGGATCCTCATTGCTCCTTCCAGATGC |
|  | AD-SINA3-F | GGAGGCCAGTGAATTCATGGACATGGACAGCGTGGAGTGC |
|  | AD-SINA3-R | CGAGCTCGATGGATCCTCAGCTACAAAGGTTCGGTATGC |
|  | AD-SINA4-F | GGAGGCCAGTGAATTCATGGACGTGGACAGCGTCGAG |
|  | AD-SINA4-R | CGAGCTCGATGGATCCTTAGCTACAGAGATTTGGTATGC |
|  | AD-SINA5-F | GGTTCCGCGTGGATCCATGGACATGGCGAGCATCGA |
|  | AD-SINA5-R | GTCGACCCGGGAATTCTCAGCTGAATAGGTTTGGAATG |
|  | AD-OsDIS1-F | GGAGGCCAGTGAATTCATGGCCTCAGTTACTTATATTG |
|  | AD-OsDIS1-R | CGAGCTCGATGGATCCTCACTGTTCCTTCCAAATTC |
|  | BD-SINA1-F | CATGGAGGCCGAATTCATGGCATCAGTTACTTATCTTG |
|  | BD-SINA1-R | GCAGGTCGACGGATCCTCACTGCTCCTTCCAAATCCTTC |
|  | BD-SINA2-F | CATGGAGGCCGAATTCATGGCCCCAGGAAGCAGCATTG |
|  | BD-SINA2-R | GCAGGTCGACGGATCCTCATTGCTCCTTCCAGATGC |
|  | BD-SINA3-F | CATGGAGGCCGAATTCATGGACATGGACAGCGTGGAGTGC |
|  | BD-SINA3-R | GCAGGTCGACGGATCCTCAGCTACAAAGGTTCGGTATGC |
|  | BD-SINA4-F | CATGGAGGCCGAATTCATGGACGTGGACAGCGTCGAG |
|  | BD-SINA4-R | GCAGGTCGACGGATCCTTAGCTACAGAGATTTGGTATGC |
|  | BD-SINA5-F | CATGGAGGCCGAATTCATGGACATGGCGAGCATCGA |
|  | BD-SINA5-R | GCAGGTCGACGGATCCTCAGCTGAATAGGTTTGGAATG |
|  | BD-OsDIS1-F | CATGGAGGCCGAATTCATGGCCTCAGTTACTTATATTG |
|  | BD-OsDIS1-R | GCAGGTCGACGGATCCTCACTGTTCCTTCCAAATTC |
| Constructs for BiFC assays | P2YC-DEP2-F | GAACGATAGTTAATTAAATGGAGCCCGACGCCCCGCTC |
|  | P2YC-DEP2-R | CACCTCCTCCACTAGTCCTGAGCCTTGCATCACCCCCCT |
|  | P2YN-SINA3-F | GAACGATAGTTAATTAAATGGACATGGACAGCGTGGAGT |
|  | P2YN-SINA3-R | CACCTCCTCCACTAGTGCTACAAAGGTTCGGTATGCAG |
|  | P2YN-SINA5-F | GAACGATAGTTAATTAAATGGACATGGCGAGCATCGA |
|  | P2YN-SINA5-R | CACCTCCTCCACTAGTGCTCAGTTGAATAGGTTTGG |
| qRT-PCR | DEP2-RT-F | AGGGCAAGCTGTTTCAAGTG |
|  | DEP2-RT-R | TGTGACAATGGTGGGCTACT |
|  | OsDIS-RT-F | CGCAATGTATCCTCCCATTCA |
|  | OsDIS-RT-R | TTACCCAGTTCATGCCTACAAG |
|  | OsSINA1-RT-F | AATGCTACCTGGATGCTCAC |
|  | OsSINA1-RT-R | CATCATCTCCCATGAACCGTAG |
|  | OsSINA2-RT-F | CTGACGGGCTTGAATGATTTG |
|  | OsSINA2-RT-R | CACCCTATGCTTGCAGTTAGA |
|  | OsSINA3-RT-F | CCCGTCTGCACCAACTCCAT |
|  | OsSINA3-RT-R | CAGGTAGGGCACCGGTTGTG |
|  | OsSINA4-RT-F | GAAGCGCAGTGCATGTTTAG |
|  | OsSINA4-RT-R | CCTCAAATGGGCAACAAGATAAG |
|  | OsSINA5-RT-F | GCTTGAGCTACCCTGCAAATA |
|  | OsSINA5-RT-R | GAGCATTCAGAGCCAGCATAA |
|  | Ubiqutin-F | GCTCCGTGGCGGTATCAT |
|  | Ubiqutin-R | CGGCAGTTGACAGCCCTAG |
